# Consensus-based reporting guideline for participatory development and evaluation of digital health interventions

**DOI:** 10.1038/s41746-026-02355-5

**Published:** 2026-01-20

**Authors:** Vera Weirauch, Anne Mainz, Julia Nitsche, Theresa Sophie Busse, Sven Meister

**Affiliations:** 1 Health Informatics, Faculty of Health, Witten/Herdecke University, Witten, Germany; 2 Department Healthcare, Fraunhofer Institute for Software and Systems Engineering ISST, Dortmund, Germany; 3 Department of Didactics and Educational Research in Health Care, Faculty of Health, Witten/Herdecke University, Witten, Germany; 4 Faculty of Health, Witten/Herdecke University, Witten, Germany

**Keywords:** Public health, Outcomes research

## Abstract

Existing literature reveals shortcomings in reporting on digital health interventions (DHIs) development and evaluation, resulting in limited traceability and hampered knowledge growth. Despite existing health research reporting guidelines, a specific guideline for the participatory development and evaluation of DHIs is lacking. This study aimed to develop a consensus-based reporting guideline to increase the transparency and comparability of both the participatory development and evaluation of DHIs. Following the methodology recommended by the EQUATOR Network and Mohers et al., a web-based Delphi Study comprising three rounds (two surveys; one workshop) was conducted. An international panel of 66 experts from 23 countries agreed on 68 items for the final reporting guideline, derived from existing reporting guidelines and refined through expert consultation. The final consensus-based reporting guideline ParDE-DHI addresses a significant gap in the systematic reporting of participatory development and evaluation of DHIs. Tailored to the unique challenges of participatory design and research, it enhances the credibility and comparability of study designs and results. This is a crucial step towards promoting best practices and advancing methodological rigor in the field. International and interdisciplinary panel input ensures adaptability and relevance across digital health contexts, ultimately fostering improved participation and knowledge sharing within the research community.

## Introduction

Nationally and internationally, healthcare faces ever greater challenges due to demographic change, increased life expectancy, and the growing multimorbidity of patients. At the same time, there is a positive trend regarding a growing desire to increase shared decision-making and patient empowerment, which also requires a realignment. Additionally, there is a shortage of qualified personnel, and a problem with the allocation of resources, and a lack of access to relevant data^1,2^. One way to meet these challenges are digital health interventions (DHIs)^2–4^. In recent years, the implementation of DHIs has increased and transformed the landscape of healthcare^3,5^. According to the classification by the World Health Organization (WHO), DHIs are understood as digital functionalities that aim to achieve health sector objectives^1^. These interventions have shown great promise in improving health outcomes, enhancing access to care, and promoting patient empowerment^2–4,6–8^.

As indicated in the literature, it is not uncommon for end-users not to utilize DHIs according to the intended purpose or to encounter difficulties regarding acceptance and adherence^9,10^. To ensure that DHIs are designed in a target group-specific and needs-based manner but can also mitigate acceptance, adherence, and usability problems, the perspectives of potential end-users and other stakeholders are indispensable^3,8–11^. These perspectives can be collected and included using participatory research and design approaches. Drawing from an interdisciplinary origin and evolution of participatory design and research approaches, they consist of a variety of overlapping collaborative methods, concepts, and frameworks^12^. Examples include participatory health research (PHR), public and patient involvement (PPI), user/human-centered design (UCD/HCD), and co-design^3,11–15^. The multiplicity of terms and overlapping methodologies frequently complicates the delineation of precise boundaries. In this article, participatory design and research are used as a generic term, with the understanding that it encompasses the following characteristics, as delineated in extant literature: mutual learning processes, the democratization of decision-making processes, the rendering of latent knowledge structures visible, and mutual creativity^14,16,17^.

To ensure and continuously increase the aforementioned potential of DHIs, it is necessary to incorporate stakeholders in the development and evaluation as part of participatory design and research approaches. In this context, development is understood as the process of creating, designing, and building a DHI, often consisting of iterative cycles and multiple stages, such as initial idea generation, prototyping, and refinement^12,14^. In the given context, evaluation is associated with the function and intent of the DHI. It focuses on assessing the DHIs value and effectiveness during or after its creation, highlighting outputs, outcomes, or impact of that DHI^10,17,18^. The provided feedback can inform (further) development, creating a cycle of continuous improvement. In participatory design and research, the process of developing and evaluating DHIs are closely intertwined and therefore should be reported jointly^10^. Stakeholders in this context can be the public, patients, healthcare providers, developers, and policymakers, who are involved in the implementation, assessment, use, or distribution of DHIs.

Unfortunately, the participatory development and evaluation of DHIs present unique methodological challenges due to the complexity and variability inherent in technological solutions, in their interdisciplinary and multisectoral nature, and in their integration into healthcare systems^3,7,8,19^. Literature indicates a broad variety and heterogeneity of incorporating stakeholders in the development and evaluation of DHIs, as well as an inadequate reporting on participatory development and evaluation of DHIs^3,4,11,19–22^. This inconsistent reporting creates a fragmented evidence base, making it difficult to compare study designs as well as study results and learn from previous studies^4,5,11,19^.

Although approaches that incorporate stakeholder involvement have gained increasing importance in healthcare, there is an absence of a reporting guideline for the participatory development and evaluation of DHIs to date. As those topics are linked together within the participatory design and research, a reporting guideline is therefore needed that takes both topics into account.

The aim is to develop and reach consensus on a guideline for the reporting of participatory development and evaluation of DHIs in the form of a checklist instrument in a process with experts from the field. The guideline is intended to work as a bridge between academic research and practice by promoting documentation and improving comparability as well as knowledge sharing.

## Results

### Panel characteristics

**Fig. 1:**
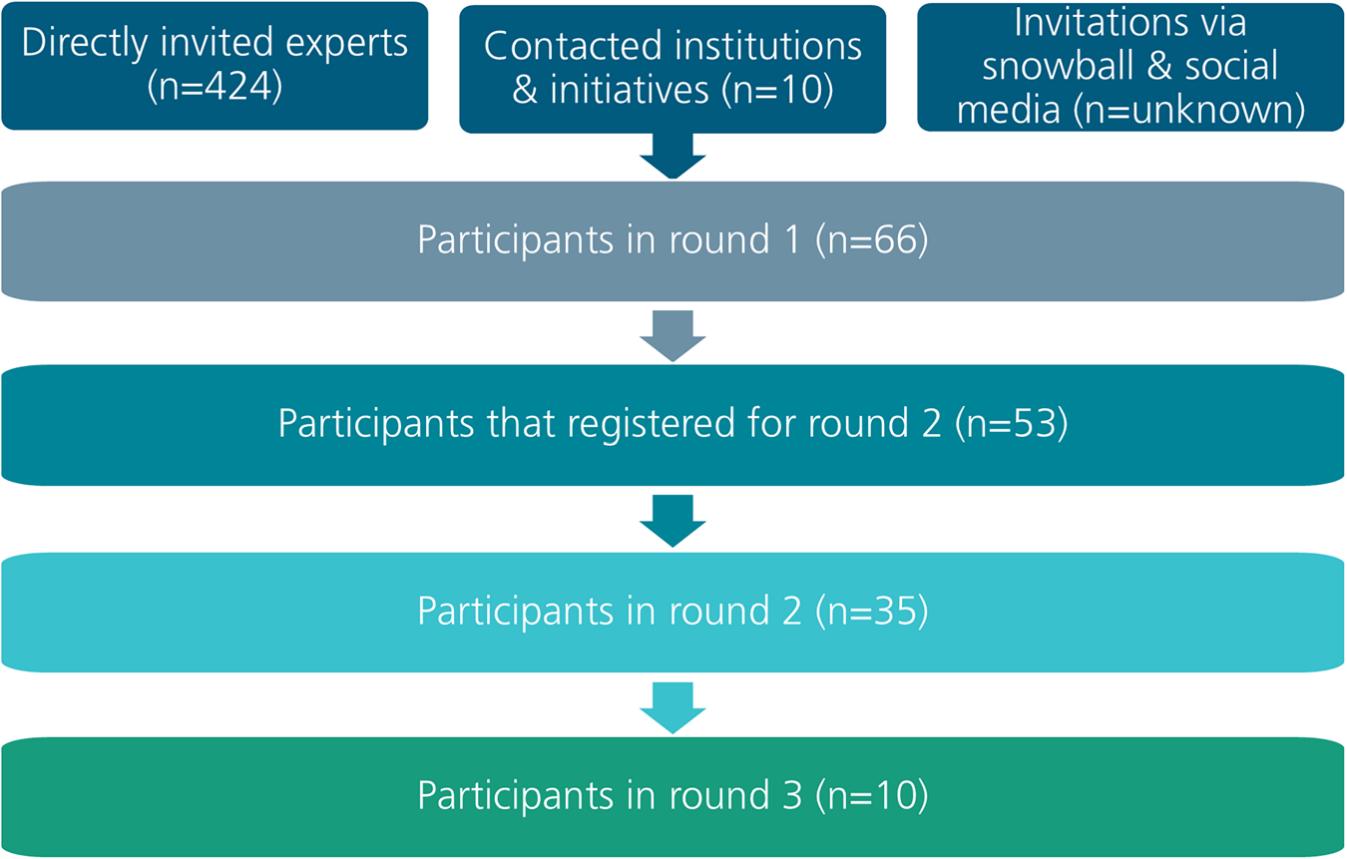
Recruitment process and study cohort size. The diagram illustrates the flow of participant recruitment across three Delphi rounds, including the different invitation pathways and subsequent participation numbers. Directly invited experts (*n* = 424), contacted institutions and initiatives (*n* = 10), and invitations via snowball sampling and social media (*n* = unknown) formed the initial recruitment pool, resulting in 66 participants in round 1. Of these, 53 participants registered for round 2, with 35 ultimately taking part in round 2 and 10 in round 3.

A total of 424 experts who had been identified as having expertise in participatory development and/or evaluation of DHIs and 10 appropriate institutions and initiatives were contacted directly. 66 individuals (15.6%) agreed to participate in the first round of the Delphi study. Of these, 53 (80.3%) expressed their written interest in also contributing to the second round and 35 (53.03%) actually participated. The final Delphi round consisted of 10 participants. A summary of the recruitment flow and the study cohort size is illustrated in Fig. 1.

Overall, the international panel includes experts from 23 different countries, whereby experts from Germany form, in every round, the biggest geographical group. Primarily, the experts have an academic background. The majority of the participants judged their experience in evaluating DHIs and incorporating stakeholders in the development and/or evaluation of DHIs as familiar or very familiar. Table 1 presents the characteristics of the panel of each round.

**Table 1:** Panel characteristics—self-reported

Characteristics	First round (*n* = 66)	Second round (*n* = 35)	Third round (*n* = 10)
**Professional Background**			
Science related to healthcare	53 (80.0%)	30 (85.71%)	8 (80.0%)
Healthcare industry	5 (8.0%)	1 (2.86%)	2 (20.0%)
Healthcare provision	5 (8.0%)	2 (5.71%)	0 (0.0%)
Others	3 (4.0%)	2 (5.71%)	0 (0.0%)
**Gender**			
Female	38 (58.0%)	20 (57.14%)	4 (40.0%)
Male	26 (39.0%)	13 (37.14%)	5 (50.0%)
Nonbinary	2 (3.0%)	2 (5.17%)	1 (10.0%)
**Birthyear**			
1951-1960	1 (1.5%)	1 (2.86%)	0 (0.0%)
1961- 1970	8 (12.3%)	5 (14.29%)	0 (0.0%)
1971- 1980	12 (18.5%)	7 (20.0%)	5 (50.0%)
1981-1990	23 (35.4%)	12 (34.29%)	5 (50.0%)
1991- 2000	20 (30.8%)	10 (28.57%)	0 (0.0%)
2001- 2010	1 (1.5%)	0 (0.0%)	0 (0.0%)
**Location**			
Australia	4 (6.1%)	4 (11.43%)	1 (10.0%)
Austria	3 (4.5%)	0 (0.0%)	0 (0.0%)
Belgium	2 (3.0%)	0 (0.0%)	0 (0.0%)
Canada	2 (3.0%)	1 (2.86%)	0 (0.0%)
China	2 (3.0%)	2 (5.71%)	0 (0.0%)
Denmark	1 (1.5%)	1 (2.86%)	0 (0.0%)
France	1 (1.5%)	0 (0.0%)	0 (0.0%)
Germany	23 (34.8%)	14 (40%)	3 (30.0%)
Greece	1 (1.5%)	1 (2.86%)	1 (10.0%)
Hungary	1 (1.5%)	0 (0.0%)	0 (0.0%)
Ireland	2 (3.0%)	0 (0.0%)	0 (0.0%)
Italy	2 (3.0%)	2 (5.71%)	0 (0.0%)
Kenya	2 (3.0%)	0 (0.0%)	0 (0.0%)
Netherlands	2 (3.0%)	1 (2.86%)	0 (0.0%)
Portugal	2 (3.0%)	1 (2.86%)	0 (0.0%)
Spain	2 (3.0%)	2 (5.71%)	2 (20.0%)
Sweden	2 (3.0%)	0 (0.0%)	0 (0.0%)
Switzerland	2 (3.0%)	2 (5.71%)	1 (10.0%)
Taiwan	1 (1.5%)	0 (0.0%)	0 (0.0%)
Tanzania	1 (1.5%)	0 (0.0%)	0 (0.0%)
Uganda	2 (3.0%)	1 (2.86%)	0 (0.0%)
United Kingdom (UK)	2 (3.0%)	2 (5.71%)	1 (10.0%)
United States of America (USA)	4 (6.1%)	1 (2.86%)	1 (10.0%)

### Expert consensus out of round 1

The experts in round 1 (*n* = 66) voted on the importance of an initial list of 64 items, of which 42 (68.75%) met the predefined consensus threshold of 75% and therefore were retained. Nevertheless, the 20 items (31.25%) that did not meet the consensus rate were close to the 75% predefined consensus, with the 4 criteria with the lowest consensus rate reaching 57.6%. A total of 192 quality comments were collected which, encompassing suggestions for revising existing content (*n* = 64), suggestions for revising scope (*n* = 40), recommendations for restructuring existing content (*n* = 12), general criticism (*n* = 3), general approval (*n* = 24) and others (*n* = 43). Following these, existing items were either revised for clarity rewording, clarification or augmenting suggestions (*n* = 30), relocated (*n* = 5), or excluded (*n* = 3) based on the qualitative feedback from the panel as well as the research team’s judgment. Furthermore, participants proposed the addition of new content, resulting in nine new items (1 in Abstract, 2 in Background, 4 in Method, 1 in Results, 1 in Others). Quality comments could be allocated to each section (Abstract to Others) to support the general approach of the Delphi study, or to contradict the omission of items (*n* = 20), which is interpreted as confirmation of the initial completeness of the guideline. The following quotations exemplify the aforementioned point: “This is an interesting Delphi (…)” (Comment 2j, Supplementary Information S4) and “I like that.” (Comment 7j, Supplementary Information S4). Detailed statistics on the consensus and distribution of each item from the first round are presented in Supplementary Information S3 and details indicating how each quality comment did inform the evolution of the items in Supplementary Information S4.

**Fig. 2:**
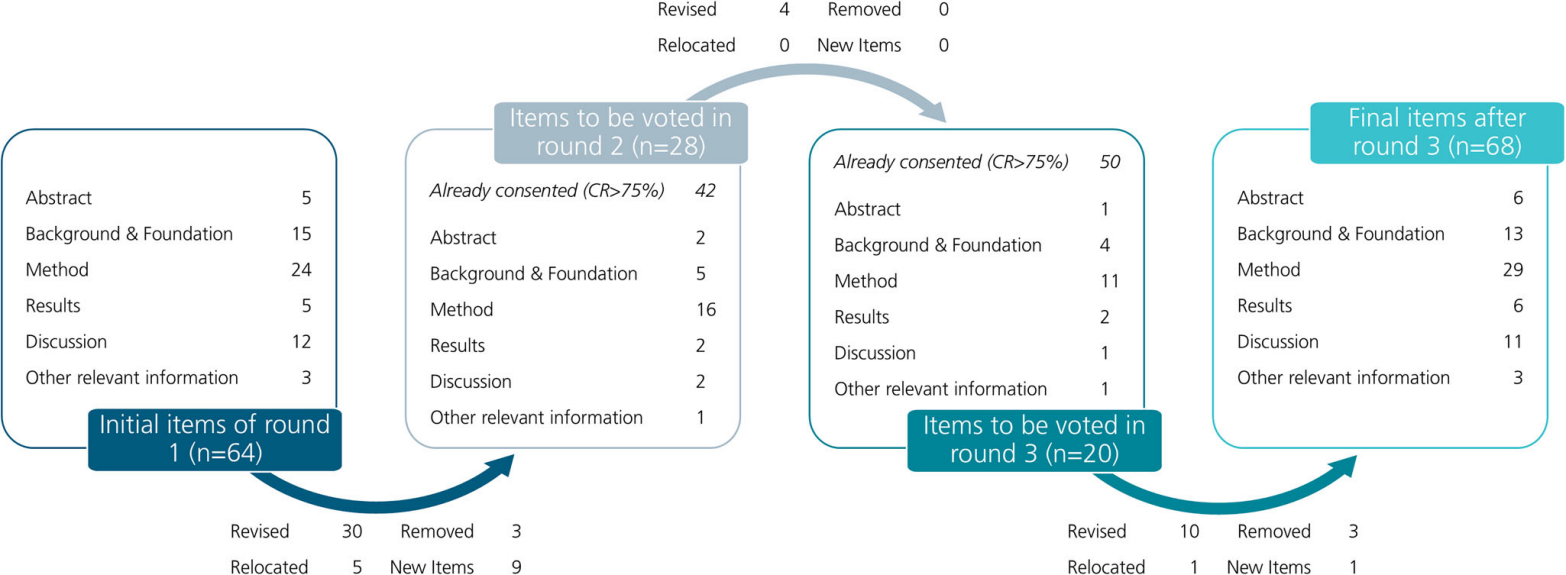
Illustration of the reporting guideline evolution in the Delphi process. Number and distribution of items across manuscript sections from the initial 64 items in round 1, through items consented or revised and those carried forward to voting in rounds 2 (*n* = 28) and 3 (*n* = 20), to the final set of 68 reporting items.

Figure 2 illustrates the evolution of the reporting guideline through the three-round Delphi study. Furthermore, Supplementary Information S2 summarizes the item ratings, enabling comparison of the rating distributions for each round.

In terms of the preferred format for the guideline, 74.24% of respondents (*n* = 49) indicated a preference for a fillable PDF or Word document. Furthermore, nearly half of the participants (48.48%, *n* = 32) preferred that examples of reference options (e.g., WHO) should be given, followed by 42.42% (*n* = 28) who had no preference, and 9.09% (*n* = 6) who declined to include reference examples. Including reference examples was justified by the statements that examples provide guidance on how to answer the question(s) and therefore improve ease of use and clarity. The following quote from the quality comments summarizes the reasons for including reference examples: “[…] Including specific examples helps to clarify the intended standard or approach, making it easier for readers to understand, contextualize, and replicate the methodology. It also lends credibility to the work by showing alignment with established and reputable frameworks.” (Comment 13.x, Supplementary Information S4).

### Expert consensus of round 2

The experts in round 2 (*n* = 35) voted on the importance of 28 items. Among these, 15 items were previously assessed with a critical rating of less than 75%, and 9 items were newly added based on the qualitative responses from the previous round. Of the 28 items, 8 (28.57%) met the predefined consensus threshold of 75%, while 20 items (71.43%) did not. In this round, a total of 26 quality comments were collected which, encompassing suggestions for revising existing content (*n* = 12), suggestions for revising scope (*n* = 5), recommendations for restructuring existing content (*n* = 3), general approval (*n* = 3) and others (*n* = 3). In consequence, four existing items were revised for clarity by rewording or augmenting suggestions. The remaining 16 items were taken over for discussion in the third round, a collaborative workshop. 34 experts indicated an interest in participating in the final round in the form of an interactive workshop.

Regarding the general rating, a total of 4 comments were categorized as “General approval” (*n* = 3) and none as “General criticism” (*n* = 0). The following quotations exemplify the general approval for the Delphi: “(…) I would be interested to read about the final research findings of this study.” (Comment 1c, Supplementary Information S6) and “I love your work.” (Comment 7b, Supplementary Information S6). Detailed statistics on the consensus and distribution of each item from the second round are presented in Supplementary Information S5 and details indicating how each quality comment did inform the evolution of the items in Supplementary Information S6.

### Expert consensus of round 3

The experts in round 3 (*n* = 10) discussed and commented on 20 items with remaining discrepancies from the former round, while also being provided with an overview of the 50 items on which consensus had already been reached. First, the discussion took place in virtual breakout rooms consisting of up to three randomly allocated individuals. Participants collected their comments on sticky notes on the digital concept board (using miro.com). Afterwards, the comments were discussed and weighted with the whole group. Seven items directly reached consensus, 10 had a rewording or augmentation, one was relocated, and three were reached to consensus to be deleted. Furthermore, one new item resulted from the discussions. The concept board with all comments can be found in Supplementary Information S7.

After incorporating the results from Round 3, the final version of the reporting guideline was created. This version comprises 68 items, including all sub-items (see Fig. 2), and is available in Supplementary Information S9. Although the Delphi process began with 64 items, the total increased to 68 by the end of Round 3. This was due not to reluctance to remove items, but to the addition of new items identified as important by panelists during the process. The final reporting guideline begins with an introductory text. This text is intended to assist in using the reporting guideline and responds to the corresponding request for an explanatory introductory text that was made several times during the entire Delphi process. A document containing explanations and elaboration (E&E) suggested by the EQUATOR network is currently under development and will be finalized subsequent to the dissemination of this paper.

## Discussion

The participatory development and evaluation of DHIs as part of a participatory design and research approach demonstrates a broad methodological variety and heterogeneity, as well as an insufficient reporting of it^4,5,11,20–22^. Following this, a pressing need for a reporting guideline that can facilitate the systematic participatory development and evaluation of DHIs and promote best practices can be identified. This extensive, international, and multifaceted Delphi study has addressed the aforementioned gap through the formulation of a consensus-driven reporting guideline. The final reporting guideline for the participatory development and evaluation of DHIs (ParDE-DHI: **Par**ticipatory **D**evelopment & **E**valuation of **D**igital **H**ealth **I**nterventions) encompasses a total of 68 items that have reached consensus upon by an international expert panel of Delphi participants. Geographical and cognitive diversity are strengths of the study. By including experts from up to 23 different countries, as well as experts with a professional background not only in health-related science but also in the healthcare industry and healthcare provision, it is assumed that the reporting guideline will promote participatory development and evaluation, as well as its documentation. Furthermore, it is expected to improve comparability and knowledge sharing and thus will be well received by the digital health community.

This reporting guideline builds on existing published work. Although the foundational scoping review was focused on participatory evaluation, the orientation and inclusion of established reporting guidelines and the active contribution of Delphi experts helped ensure that both participatory development and evaluation elements were adequately represented in the final guideline. Through the orientation towards established reporting guidelines and the extraction as well as adjustment of several items with the specific context, it is ensured that the newly developed guideline is both comprehensive and known in use. By leveraging proven elements from existing guidelines, the new reporting guideline benefits from validated guidelines, enhancing its credibility and facilitating its adoption within the scientific community. By tailoring the items to address the unique requirements and challenges of participatory development and evaluation of DHIs, the requirements and framework conditions of the literature concerning the reporting of participatory design and research were considered. This includes aspects such as a definition of participatory approaches, explanation of the context of the participation, explanation of the level of participation, and explanation of the evaluation method, including references to existing frameworks as well as definitions of evaluation criteria^12,21,23^. All in all, this approach not only enhances the credibility and acceptance of the guideline within the research community but also ensures that essential aspects are comprehensively addressed while allowing for the necessary contextual adaptations relevant to participatory design and evaluation of DHIs.

ParDE-DHI provides a set of recommendations on information that should be included for a comprehensive reporting of participatory development and evaluation of DHIs. This also includes guidance on how some information might best be presented and lists of possible frameworks for reference. The reporting guideline is intended for use by all individuals engaged in participatory development and/or evaluation of DHIs. However, it may be necessary to consider additional reporting guidelines in conjunction with ParDE-DHI (e.g., TIDieR checklist for intervention description, GUIDED checklist for reporting intervention development studies or others). The decision regarding the allocation of supplementary reporting guidelines is at the discretion of the researchers^24,25^. The inclusion of example references, such as the World Health Organization (WHO), ensures the adaptability of the reporting guideline to individuals with varying degrees of expertise in the domain of participatory design and research of DHIs. Nevertheless, the selection of appropriate formats and frameworks is up to the researcher and can also be something that is not listed as an example.

In contrast to the tendency of some Delphi-guided guideline developments to predominantly reduce the item pool, our Delphi resulted in a slight increase in the number of items. This adaptive strategy, which permitted both the elimination and addition of items, enabled the panel to fill gaps and increase the guidelines’ relevance and completeness. It is acknowledged that this approach, while less parsimonious, prioritizes comprehensive and practical coverage over item reduction per se.

The incorporation of open-ended qualitative feedback served to enrich the quantitative Delphi data, providing nuanced rationale for the refinement of content and scope. The thematic insights facilitated iterative enhancement of the guideline’s conceptual structure, thereby ensuring that expert perspectives were meaningfully integrated into each revision step. In the qualitative comments and via e-mail, there was considerable support for (1) the need for and plan to create a reporting guideline for the participatory development and evaluation of DHIs, and (2) the methodological approach to its creation. Therefore, we assume the reporting guideline will be received well by the scientific community and will improve the conduct, reporting and replicability of participatory studies. Nevertheless, it is essential to disseminate the reporting guideline effectively and to ensure that the target fields are thoroughly familiarized with it. In terms of dissemination, the reporting guideline is registered as in development in the EQUATOR network. In addition, all experts will be apprised of the final guideline and will be asked to utilize the reporting guideline. It is hoped that this will support dissemination, provide feedback and insight into the use of the guideline.

The limitations of the present study are associated with various aspects that are inherent to consensus studies. A strict predefined consent threshold of 75% was selected for this Delphi study, which results in items being either kept, updated and re-evaluated or rejected. Inspired by CONSORT-EHEALTH checklist (V.1.6.1), iCHECK-DH and the discussion of DELPHISTAR, it could have been possible to consider dividing the items into different categories based on the consent rate, e.g., into three categories with (I) highly consensual/recommended/mandatory (e.g., CR 75% and above), (II) essential (e.g., CR between 60% and 75%) and (III) possible inclusion depending on study context/nonmandatory (e.g., CR less than 60%)^5,6,26^. Adopting this strategy could have yielded not only a more nuanced but also a more complex reporting guideline.

Although the desired geographical and cognitive diversity of the expert panel was successfully achieved, the presence of biases within the panel is a potential concern, given the predominance of experts with health science backgrounds (exceeding 80% in each Delphi round) and a predominance of experts hailing from Germany. Potential explanations for these imbalances may include the composition of the German author team, as well as the issue that the intended reporting guideline primarily affects individuals working in the health sciences. Sex- and gender-based analyses were not conducted, as the Delphi process focused on expert consensus regarding reporting guidelines rather than participant demographics. We acknowledge this as a limitation and recommend that future research explore potential sex and gender influences in reporting guidelines for digital health interventions.

A further limitation is the absence of citizen and patient representatives who have participated in participatory development and/or evaluation of DHIs. While the composition of the panel concentrated on experts with methodological or practical expertise in this context, the approach excluded experiential knowledge from public contributors. It is acknowledged that citizens possess a unique perspective on this matter. Consequently, future research could investigate the participation preferences and motivation of citizens in the development and evaluation of DHI, as well as the reporting of this.

The general response rate and the decreasing participation rate per round are consistent with the expected outcomes resulting from similar Delphi studies. Possible reasons for non-participation could include not receiving the emails, language barriers, or, especially in the case of the third round, different time zones. Consequently, specialists with a high level of expertise and experience in this area may have been excluded. To address this issue, reminder emails were disseminated, and the third-round workshop was scheduled on two separate dates. Additionally, the concept board was made available for comment independent of the workshop dates.

To conclude, the development of the consensus-based reporting guideline for the participatory development and evaluation of DHIs (ParDE-DHI) marks an advancement in the field of digital health research. By addressing the methodological gaps identified in existing literature, this guideline provides a structured guideline that enhances the transparency and comparability of participatory development and evaluation. The involvement of a diverse international expert panel ensures that the guideline is robust, adaptable, and relevant across various contexts in healthcare. Through the integration of elements from established reporting guidelines, the new reporting guideline leverages validated methodologies, thereby enhancing its credibility and facilitating adoption within the scientific community. The guideline’s emphasis on comprehensive reporting of participatory processes not only fosters improved stakeholder engagement but also contributes to democratizing development and research in the field of digital health. Effective dissemination and implementation of this guideline will be crucial to its success. The registration with the EQUATOR Network and engagement with experts aim to promote widespread adoption and feedback. This initiative aims to enhance the quality and impact of participatory studies within the digital health domain, ultimately supporting enhanced healthcare outcomes and public as well as patient empowerment.

## Methods

### Overview

To develop a consensus-based guideline for the reporting of the participatory development and evaluation of DHIs, this study follows the methodology recommendations of the EQUATOR Network for the development of reporting guidelines and is oriented on Mohers et al. Guidance for Developers of Health Research Reporting Guidelines^27^. Following these, the consent-creating Delphi method was chosen as a central element of the creation process.

A three-stage Delphi study was conducted by a multidisciplinary team of representatives from relevant fields (VW, AM, JN, SM, TSB). VW is a doctoral researcher for health sciences, AM is a doctoral researcher for health informatics, JN is a postdoctoral researcher from health didactics with a background in business psychology, SM is a professor for health informatics, and TSB is a professor for digital health with a background in health sciences. The expertise of these includes high familiarity with participation, development, and evaluation, as well as reporting and publishing these in the context of digital health. VW prepared the two online surveys as well as the workshop with support from AM, JN, and TSB. The evaluation of the previous round was consulted in each case to continuously refine the reporting guideline. VW associated information material and organized the digital “Face-to-Face Consensus Meeting“ in the form of a collaborative workshop with support from AM, JN, and TSB. After the workshops, VW finalized the reporting guideline and obtained further feedback from AM, JN, and TSB regarding wording and form. SM supervised the process.

**Fig. 3:**
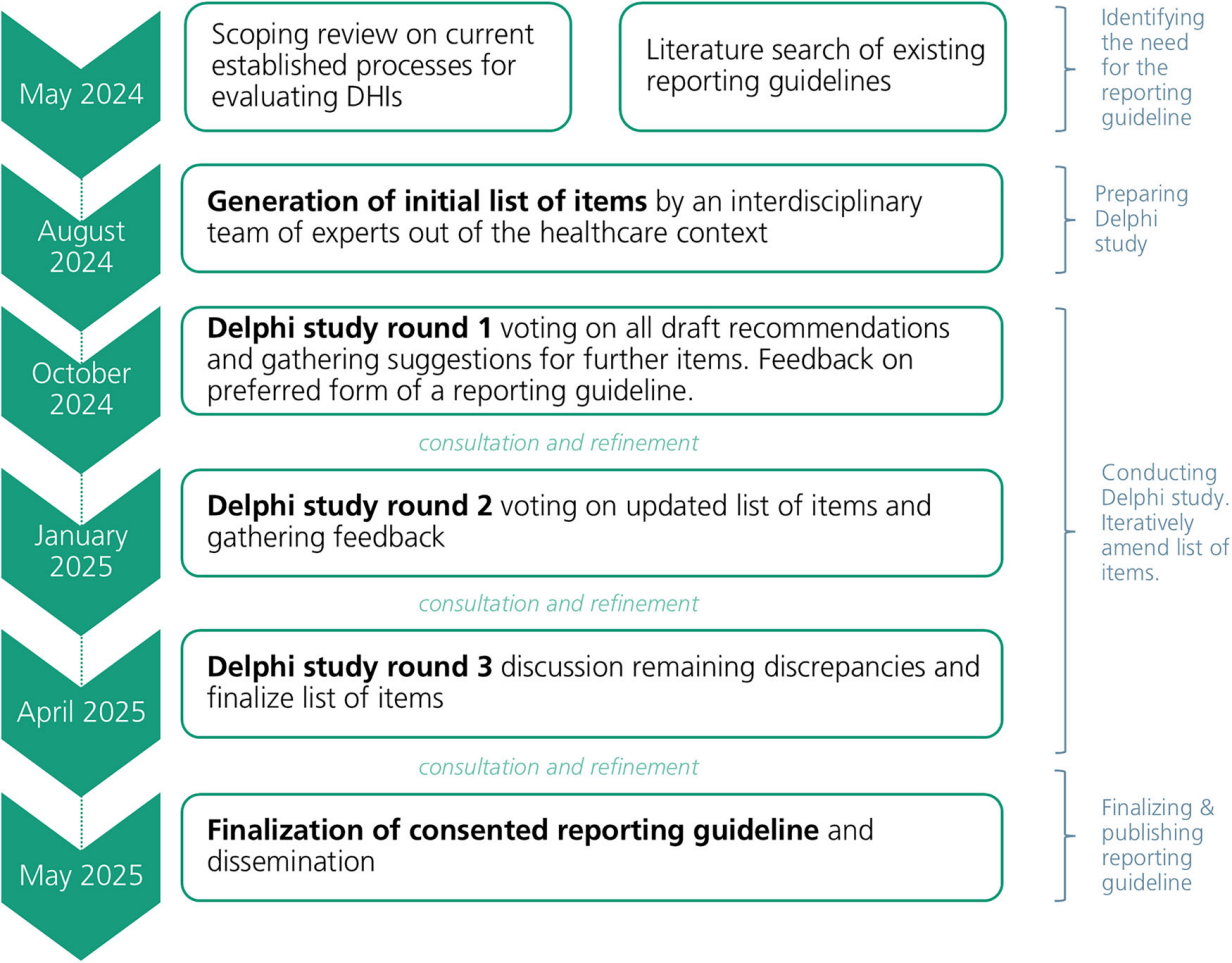
Overview of the study design. Timeline of key project phases from May 2024 to May 2025, including the scoping review and literature search, generation of the initial item list, three Delphi rounds with iterative consultation and refinement, and the finalization and dissemination of the consented reporting guideline.

Figure 3 provides a visual representation of the study’s process and time frame.

To ensure quality and comparability, the study adheres to the reporting guideline “Delphi studies in social and health sciences–recommendations for an interdisciplinary standardized reporting” (DELPHISTAR)^26^.

A study protocol was registered a priori with the Center for Open Science (OSF)^28^. The preregistered study protocol primarily referenced participatory evaluation; however, the final consensus process encompassed participatory development and evaluation. The adoption of this was motivated by the necessity to comprehensively capture the participatory lifecycle of DHIs, thereby acknowledging the inherent overlap and intertwined nature of these two components. Furthermore, the reporting guideline has been registered in the EQUATOR Network register of reporting guidelines under development^29^.

### Development of the initial draft of the intended reporting guideline

Prior to the Delphi survey, the multidisciplinary author team (VW, AM, JN, SM, TSB) generated a list of items for consideration in an iterative process. VW prepared a preliminary list of items informed by a (1) prior scoping review and (2) existing reporting guidelines. Recognizing that this scoping review focused on participatory evaluation of DHIs for public end users and therefore did not fully capture participatory development aspects, subsequently an additional screening of existing key reporting guidelines is conducted^21^. For the screening of existing key reporting guidelines database and repository searches were performed in the EQUATOR Network and JMIR’s e-collection/theme issue “Digital Health Reporting Standards, Quality & Transparency in e-health” using keywords including “reporting guideline,” “checklist,” “digital health intervention,” “digital health,” and “participat*”. Key reporting guidelines (such as CHEERS, CONSORT-EHEALTH, COREQ, GIRPP2-LF, mERA, STROBE) and newly developed guidelines by exploring papers that are following the JMIR e-collection/theme issue “Digital Health Reporting Standards, Quality & Transparency in e-health” (such as iCHECK-DH) were screened^4–6,11,30–32^. Supplementary Information S1 provides an overview of all guidelines from which items were taken. During this process, several items were extracted, merged, and adjusted to align with the specific context. VW, AM, JN, and TSB discussed the initial list of items for consideration in a consensus meeting and previous individual work. They adapted it, shortened it, and expanded it in various areas. The final draft of the reporting guideline under consideration comprised 64 items, including potential subitems, distributed across six specific sections: abstract, background and foundation, method, results, discussion, and others. This draft served as an initial reporting guideline and formed the basis for the Delphi process

### Selection and recruitment of participants

For the Delphi study, concerning the composition of participants, attention was paid to cognitive diversity, which means that the aim was to systematically bring together the specialized and decentralized knowledge of experts from academia with the knowledge and real-world experience of experts from the health industry and practice. Considered experts were people with expertise in the participatory development and/or evaluation of DHIs. This includes scientists related to healthcare (e.g., health services research, health or medical informatics, human-machine interaction), employees in the healthcare industry (e.g., health software developers or service providers, digital health startups), or in healthcare provision (e.g., physicians, nursing staff, medical assistants). Eligibility as an expert was defined by meeting at least one of the following criteria: (1) authorship of at least one peer-reviewed publication related to participatory design and research in context of digital health; (2) a professional position involving the conceptualization, development, evaluation, or implementation of DHIs; or (3) recognized engagement in relevant digital health or participatory research networks. This inclusive definition aimed to capture both academic and applied expertise in line with existing methodological recommendations for Delphi studies. Also, to ensure geographical and cultural diversity among participants, no geographic limitations were imposed, and a representation of at least 10 different countries for the first two rounds was aspired.

In general, there are no agreed standards about the panel size of classic Delphi studies^33,34^. Based on other Delphi studies in the healthcare context, the authors have targeted a sample size of at least 50 individuals for the first round^2,26,35,36^.

To identify potential participants, a mixed sampling strategy was used. Primarily, potential participants were identified via their publications or professional positions. The data extraction of a previously conducted scoping review served as a basis because it identified publications that describe evaluation processes of DHIs primarily aimed at public end users^21^. These experts identified through purposeful sampling were invited directly via email, gathered from the publicly available contact pages. Additionally, context-relevant initiatives, networks, and associations were addressed, and invitations were shared through social media (e.g., LinkedIn).

Each invitation comprised a one-pager and a textual description, which included information regarding the rationale behind the study, the methodology to be applied, the justification for the selection of experts to participate, and a link to the questionnaire platform. Moreover, all invitations included a recommendation for snowball sampling, thereby affording experts the opportunity to suggest additional experts from their network. The participation was voluntary and anonymous. Before starting each round, informed consent was obtained from all of the participants. They were required to read and agree to the privacy policy, information about participation, and consent form before the beginning of the study.

### Delphi procedure and data collection

The Delphi technique has been established in the health sector to systematically synthesize explicit and implicit knowledge and to create consensus^34^. Findings can be used to improve evidence and acceptance of standards or guidelines^34^. The classic Delphi process has been chosen and consists of several rounds in which experts iteratively assess certain issues by answering standardized questionnaires. In the recurring rounds, the knowledge gained in the previous round is made available, so that it can be reconsidered and revised if necessary^34^. This Delphi study consisted of three rounds to iteratively create consensus. All rounds were conducted in English to facilitate international participation and therefor to ensure geographical and cultural diversity among participants.

The first two rounds were conducted in the form of online surveys using the survey tool LimeSurvey, Version 6.6.6. Before starting the first round, a pre-test consisting of 10 academics of the authors’ professional network was conducted to reduce the risk of misinterpretation of statements and instructions. These persons have expertise in the context of digital health and do not belong to the research team.

In the first round, participants scored and commented on the initial 64 items. The proposed draft of the reporting guideline was queried using standardized items on a 5-point Likert scale (“1 = very unimportant, 5 = very important” or “1 = strongly disagree, 5 = strongly agree”). The participants were queried regarding the degree of certainty associated with their judgments (“1 = extremely uncertain, 5 = absolutely certain”) at the conclusion of each section, as proposed by Nieberger et al.^26^. Additionally, they had the opportunity to propose their own items related to participatory development and evaluation for voting in subsequent rounds and were also able to provide anonymous free-text comments in general. To provide a description of the sample, the survey also contained socio-demographic inquiries (e.g., gender, age, country of residence) and questions about the participants’ expertise (e.g., professional activities, experience with participatory evaluation and/or research). The survey was open for four weeks, spanning October and November 2024. After this period, the comments were evaluated and integrated into the subsequent questionnaire by VW. If necessary, semantics were modified to enhance clarity and concision. Items failing to achieve the predefined consensus rate (CR) were revised or discussed within the authors’ teams in order to remove them or to retain them for further evaluations. According to similar Delphi procedures, consensus on a statement was considered as reached if at least 75% of participants provided a scale value of 4 (agree) or 5 (strongly agree)^26,33,34,36^.

The *second round* also entailed an online survey, which was structurally analogous to the survey administered in the first round, including scale, socio-demographic questions, and open-ended feedback fields, among other components. The survey was open for four weeks in January 2025. Participants rated the updated questionnaire, consisting of items that did not reach the predefined consensus threshold, updated items, and new items suggested by the participating experts of the first round. Changes in wording and new items were highlighted. Again, the participants were able to provide anonymous free-text comments. Furthermore, as typical for classic Delphi studies, the participants received feedback on the statistical group response from the first round for each item and a summary of the arguments made in the open-ended responses.

The *third round* was conducted as a virtual collaborative workshop. The objective of this 60-minute workshop was to address and resolve any inconsistencies that remained after the previous rounds. To ensure the participation of a broad spectrum of individuals, the virtual collaborative workshop was scheduled on two separate dates to maximize attendance. Scheduling for these workshops was conducted through a scheduling survey in which interested individuals indicated their availability. The basis of the workshops was a concept board, an interactive digital collaboration platform that permits simultaneous viewing, commenting by adding anonymous sticky notes, and organizing Delphi items and feedback, and consisting of three parts. The initial segment entailed the responses to sociodemographic inquiries and an explanation of the concept board’s functionality. The second part constituted the primary segment of the workshop, wherein the discussion and resolution of discrepancies were prioritized, with a particular focus on items below a CR of 75%. Hereby, participants had an overview of the so far consensus reached items and as bevor participants received feedback on the statistical group response for each item. In the concluding part, participants were given the opportunity to raise any issues that should be addressed in the final report and to offer additional comments. Subsequent to the conclusion of the workshops, the concept board remained open for nearly two more weeks and was shared with all individuals who had expressed interest in participating in the third round during the second round.

### Analysis

Statistical analysis was performed using SPSS (Version 29.0) and Microsoft Excel. The responses to the standardized questions were descriptively analyzed (mean, median, standard deviation, variance). A consensus threshold was defined a priori as at least 75% of respondents voted an item as “important” or “very important”. The consensus threshold was derived from the results of analogous Delphi studies conducted in the healthcare sector^26,35,36^. The open-ended responses from the text boxes were exported verbatim from the survey interface and analyzed qualitatively oriented on thematic analysis. Therefore, all qualitative comments were classified into 6 categories by VW: (1) revising existing content including rewording, clarification or augmenting suggestions for existing items; (2) revising scope including new item and shortening suggestions; (3) General criticism; (4) General approval; (5) Restructuring existing content including resorting and merging of items; and (6) Others. Coded comments were discussed by four independent researchers (VW, AM, JN, TSB) to avoid biases and to reach consensus on whether and how to incorporate changes before the next Delphi round.

The present study did not specifically address sex-based and gender-based analyses, because it aimed to gather methodological and practical expertise from a diverse, international panel of professionals rather than analyzing data related to participants’ sex or gender.

### Ethics

Ethics approval has been obtained by the Ethics Committee of Witten/Herdecke University, Witten, Germany (S-115/2024). All individuals provided voluntary, informed, and written consent to participate in the study and have the results published in a peer-reviewed article. The primary data collection approaches adhered to established ethical standards in scientific research as well as data protection regulations (General Data Protection Regulation).

## Supplementary information

Updated_Supplementary-Information_clean-Version

## Data Availability

The datasets generated from this review are available from the corresponding author upon request.
