# Supplementary material for: Consensus-based reporting guideline for participatory development and evaluation of digital health interventions
Source: NPJ Digit Med. 2026 Jan 20;9:169. doi: 10.1038/s41746-026-02355-5 (PMC12913994; doi:10.1038/s41746-026-02355-5)
Supplement: Supplementary file 1 — Updated_Supplementary-Information_clean-Version [file 41746_2026_2355_MOESM1_ESM.pdf]

# Supplementary Information

This supplementary information formed part of the original submission.

Supplement to: [Submitted paper citation.]

# Table of Contents


Supplementary Information S1: Background of Initial Draft ........ 3

Supplementary Information S2: Results Overview ........ 4

Supplementary Information S3: Detailed Results 1. Round ........ 10

Supplementary Information S4: Qualitative Results 1. Round ........ 21

Supplementary Information S5: Detailed Results 2. Round ........ 32

Supplementary Information 6: Qualitative Results 2. Round ........ 37

Supplementary Information S7: Results 3. Round ........ 39

Supplementary Information S8: DELPHISTAR Checklist ........ 41

Supplementary Information S9: Final Reporting Guideline ........ 43

References ........ 47

Supplementary Information S1: Background of Initial Draft

| Acronym | Title | Short Description | Extracted Items |
|---|---|---|---|
| A sociotechnical framework to assess patient-facing eHealth tools | A sociotechnical framework to assess patient-facing eHealth tools | A framework focusing on sociotechnical aspects of patient-facing eHealth tools | 7 |
| CHEERS | Consolidated Health Economic Evaluation Reporting Standards | Checklist focusing on health economic evaluation. primarily intended for researchers reporting economic evaluations for peer-reviewed journals and the peer reviewers and editors assessing them for publication | 8 |
| Cherries | Checklist for Reporting Results of Internet E-Surveys | Checklist focusing on Web Surveys. Similar to CHEERS, except a different focus | 6 |
| CONSORT-EHEAHLTH | consolidated Standards of Reporting Trials of Electronic and Mobile HEalth Applications and onLine TeleHealth | Checklist instrument focusing on RCT in a digital context/ e-health & m-health trials. Checklist is intended for authors of randomized trials evaluating web-based and Internet-based applications/ including mobile interventions, electronic games (including multiplayer games), social media, certain telehealth applications, and other interactive and/or networked electronic applications. | 7 |
| COREQ | Consolidated criteria for reporting qualitative research | formal checklist for reporting of qualitative studies, especially for in-depth interviews and focus groups, the most common methods for data collection in qualitative health research. | 6 |
| GREET | Development and validation of the guideline for reporting evidence-based practice educational interventions and teaching | Reporting of educational interventions for evidence-based practice (EBP) | 0 |
| GRIPP2 | Guidance for Reporting Involvement of Patients and the Public Version 2 checklist | Checklist for reporting patient and public involvement (PPI) in health and social care research | 24 |
| iCHECK-DH | Guidelines and Checklist for the Reporting on Digital Health Implementations | Checklist instrument focuses on implementation. Checklist to improve the completeness of reporting on digital health implementations. | 3 |
| mERA | mHealth Evidence Reporting and Assessment | The checklist provides guidance for complete and transparent reporting on studies evaluating and reporting on the feasibility and effectiveness of mobile health (mHealth) interventions. No focus on design, implementation or research method. | 2 |
| Murray | Evaluating digital health interventions: key questions | Research-driven approach that aims to examine evaluation challenges and outline an evaluation strategy in terms of the Research Questions (RQs) needed to appraise DHIs | 11 |
| PRISMA | Preferred Reporting Items for Systematic reviews and Meta-Analyses | Checklist for Reporting systematic reviews and meta-analyses. | 0 |
| STARE-HI | Statement on reporting of evaluation studies in Health Informatics | Guideline for writing evaluation reports in Health Informatics | 5 |
| STROBE | STrengthening the Reporting of OBservational studies in Epidemiology | Checklist for reporting observational research like cohort, case-control, and cross-sectional studies | 7 |
| GUIDED | Guidance for reporting intervention development studies in health research (GUIDED): an evidence-based consensus study | Checklist for reporting intervention development studies in health research | 0* |

** Following reviewer feedback, the GUIDED checklist has been incorporated into this list after the Delphi process. All relevant aspects of GUIDED can be also found in the final reporting guideline.*

## Supplementary Information S2: Results Overview

Abbreviations: CR= Consensus Rate (Participants who rated 4 or 5); SD= Standard Deviation; pc= previously consented (item not re-rated)

| | | | 1.Round | | | | 2.Round | | | |
|---|---|---|---|---|---|---|---|---|---|---|
| **ID** | **Item- Title** | **Item- Full Text** | **n** | **CR (%)** | **Mean** | **SD** | **n** | **CR** | **Mean** | **SD** |
| Abstract | | | | | | | | | | |
| 65 | Background | Briefly state the background and context of the study. including the state of the art and the research gap. | - | - | - | - | 35 | 77.1 | 4.00 | 1.000 |
| 1 | Aim | State the aim of the participatory evaluation and development. | 66 | 90.9 | 4.56 | 1.083 | - | pc | - | - |
| 2 | Method | Describe the key methods for evaluation and participation. such as evaluated criteria & indicators. target group. geographic place of data collection. degree of participation. | 66 | 86.4 | 4.45 | 1.040 | - | pc | - | - |
| 3 | Results | Report the key findings and their relevance as well as sample size and sociodemographic characteristics of participants. | 66 | 86.4 | 4.42 | 1.024 | - | pc | - | - |
| 4 | Conclusion | Summarize the main conclusions of the study and potential implications. | 66 | 86.4 | 4.45 | 0.948 | - | pc | - | - |
| 5 | Keywords | List relevant keywords that represent the core topics of the study. | 66 | 62.1 | 3.67 | 1.141 | 35 | 68.6 | 3.83 | 1.224 |
| Background & Foundations | | | | | | | | | | |
| 6 | Scientific Background | Describe the scientific background of the study and indicate which research was carried out in advance and which databases and terms were used to find information. | 66 | 84.8 | 4.29 | 0.924 | - | pc | - | - |
| 7 | Study Context | Describe the context of the study and the addressed problem. Describe whether the study is part of a larger research. development or implementation project. Mention from which perspective (if any) the study is performed. | 66 | 86.4 | 4.33 | 0.900 | - | pc | - | - |
| 8 | Rationale | Describe the motivation and specific reasons for the study (scientific interest. justification for expenditure. insight into problems like potential unintended side effects. addressing open research questions). | 66 | 87.9 | 4.44 | 0.879 | - | pc | - | - |
| 9 | Ethical Vote & Considerations | Please mention the ethical vote for the survey and where it was obtained. Add information about the consideration of the possibility of harm as well as the assessment of the likelihood of risks as well as adverse outcomes. | 66 | 77.3 | 4.11 | 1.242 | - | pc | - | - |
| 10 | Theory of Participation Concept | Report the definition of participation used in the study and the belonging concept (e.g.. PPI. PHR..). Describe why this definition fits your study. | 66 | 80.3 | 4.26 | 1.042 | - | pc | - | - |
| 66 | DHI Setting | Describe the setting of the DHI and the real-world context where it will be later on implemented. Add information on the health domain it addresses. the specific medical issue it targets and the intended use setting.<br>DHI Development | 66 | - | - | - | 35 | 91.4 | 4.26 | 1.039 |
| 67 | DHI Development | Describe how the DHI was developed and whether a specific framework or theory (such as behavioral change frameworks) was followed in the development process. If applicable. add a description of the history/development process of the DHI and previous formative evaluations (e.g. focus groups. usability testing) to the appendix. Describe formative research and/or content and/or usability testing with target group(s) clearly identified. as appropriate. If possible. it should be mentioned what influence the findings of the study have. | 66 | - | - | - | 35 | 74.3 | 4.00 | 1.057 |

| | | | | | | | | | | |
|---|---|---|---|---|---|---|---|---|---|---|
| | DHI Description | Describe the type of the DHI that is the object of the study by …<br>(If there is one or more comparators all the following points also have to be addressed) | | | | | | | | |
| 15 | DHI Description | a) referring to established frameworks or classification systems (e.g. those provided by the WHO/NICE) to classify DHI | 66 | 59.1 | 3.47 | 1.619 | 35 | 42.9 | 3.37 | 1.215 |
| 16 | DHI Description-Key Components | b) Listing the characteristics and key components of the DHI. Outline its primary functions as well as its user interface. Describe how the compo-nents interact with each other and which components impact on which predicted outcomes. | 66 | 80.3 | 4.30 | 1.136 | - | pc | - | - |
| 17 | DHI Description-Fit to target group | c) Describing which target group the DHI addresses and why it is likely to reach this population as well as why the population will be likely to use it. | 66 | 83.3 | 4.30 | 1.136 | - | pc | - | - |
| 18 | DHI Description-Access for vulner-able populations | d) outlining to what extent the DHI takes diversity of potential end-users and affected people into account (e.g.. adjustment of text size. text to voice. colorblind color scheme adjuster. specificity to use by minors. of-fline features. left and right-handed options) and make explicit any equity concerns that may have arised in their attempt to take the end-user into account. Outline how the equitable usage of potential end-users is con-sidered. | 66 | 81.8 | 4.18 | 1.136 | - | pc | - | - |
| 19 | DHI Description-Updating and re-visions | e) mentioning the iterations of the DHI or describing whether the interven-tion underwent major changes during the evaluation process or whether the development and/or content was "frozen" during the trial. | 66 | 69.7 | 3.80 | 1.315 | 35 | 62.9 | 3.63 | 1.031 |
| 20 | Tailoring during use | f) describing dynamic components (e.g. news feeds. changing content. usage of ongoing feedback based on the end-user's state and activities. personalization) which may have an impact on the replicability of the in-tervention. Describe whether there are any strategies to support tailoring the DHI to participants over time. | 66 | 71.2 | 3.68 | 1.349 | 35 | 65.7 | 3.69 | 1.278 |
| Method | | | | | | | | | | |
| 21 | Study design | Present key study design (participation & evaluation). including the men-tion of a possible pre-registration | 66 | 87.9 | 4.53 | 0.898 | - | pc | - | - |
| 22 | Study flow | Visualize the research process (e.g. CONSORT flowchart or others appro-priate to iterative participatory approaches). | 66 | 71.2 | 3.83 | 1.319 | 35 | 68.6 | 3.94 | 1.056 |
| 23 | Informed consent | Describe the informed consent process by where the participants were told the length of time of the study. which data were stored and where and for how long. who the investigator was. and what the purpose of the study is. Add information about the possibilities of drop-out. | 66 | 74.2 | 4.00 | 1.190 | 35 | 62.9 | 3.83 | 1.150 |
| 11 | Participation Con-cept | Describe what methodological orientation was stated to underpin the study (e.g. grounded theory. discourse analysis. ethnography. phenome-nology. content analysis). | 66 | 72.7 | 4.12 | 1.074 | 35 | 94.3 | 4.46 | 0.817 |
| | Participatory Evaluation Method | Explain the level or nature of participation and participatory evaluation methods used at various stages of the study by … | 66 | | | | | | | |
| 29 | Participatory Evaluation Method | a) defining the used evaluation criteria including their underlying indica-tors by referring to existing frameworks (e.g. WHO. NICE. EXPH. the Swiss Evaluation framework by Kowatsch et al. the approach of Murray et al. or others). | 66 | 62.1 | 3.56 | 1.469 | 35 | 57.1 | 3.51 | 1.380 |

| | | | | | | | | | | |
|---|---|---|---|---|---|---|---|---|---|---|
| 25 | Participatory Evaluation Method | b) describing and referencing the participatory methods used (e.g., co-design workshops. user testing. or participatory data analysis). | 66 | 95.5 | 3.64 | 0.715 | - | pc | - | - |
| 26 | Participatory Evaluation Method | c) describing the level or nature of participation used at various stages of the study. Refer to established theories or (stage) models of participation (e.g., Wright's et al. Stage Model of Participation. Arnstein's Ladder of Citizen Participation. Treseder's Degrees of Involvement. Shier's Pathways to Participation. Community-Based Participatory Research or others). | 66 | 75.8 | 4.02 | 1.130 | - | pc | - | - |
| 28 | Participatory Evaluation Method | d) describing the reasons behind the chosen evaluation methods and the level of evidence of the method. | 66 | 78.8 | 4.21 | 0.985 | - | pc | - | - |
| 30 | Participatory Evaluation Method | e) if established methods. frameworks or approaches are deemed inappropriate or inapplicable for the specific context of the DHI being evaluated. explaining the reasons for the exclusion briefly. | 66 | 71.2 | 3.83 | 1.210 | 35 | 57.1 | 3.51 | 0.981 |
| 27 | Participatory Evaluation Method | f) describing any approaches to participate or engage potential end-users and other affected people (e.g. patients. public. communities. clinicians or others) in the design of the study. If applicable. how is the equitable participation of all relevant people. including people in vulnerable situations considered | 66 | 81.8 | 4.14 | 0.975 | - | pc | - | - |
| 31 | Participatory Evaluation Method | g) outlining how it was ensured that vulnerable diversity in potential end-users and other affected people were adequately considered and represented in the participatory evaluation. | 66 | 77.3 | 4.03 | 1.123 | - | pc | - | - |
| 24 | Impacts/Effects of participation | If applicable. report the methods used to explore the impact of participation as well as barriers and facilitators of participation in the study. | 66 | 68.2 | 3.94 | 1.175 | 35 | 80.0 | 4.06 | 9.938 |
| 74 | Usage of AI | Report if AI was used to analyze data. | | - | - | - | 35 | 60.0 | 3.57 | 1.441 |
| | Data Collection & Measurements | Provide details on the data collection methods used for each variable of interest. by | | | | | | | | |
| 32 | Data Collection & Measurements | a) describing all relevant aspects of applied data collection methods. (e.g. questionnaire. interview. focus groups. observation. task-/scenario-completion. log file analysis). | 66 | 89.4 | 4.62 | 0.718 | - | pc | - | - |
| 33 | Data Collection & Measurements | b) explaining which roles established measurements play in the study. If established measurements can't be used. explain why. | 66 | 71.2 | 3.83 | 1.272 | 35 | 62.9 | 3.80 | 0.933 |
| 34 | Data Collection & Measurements | c) If applicable. adding a detailed description of instrument development and testing (e.g., validity. reliability. feasibility. acceptability. responsiveness. interpretability. appropriateness. precision) of newly designed measurements to the appendix. | 66 | 78.8 | 4.17 | 1.017 | - | pc | - | - |
| 35 | Data Collection & Measurements | d) describing the comparability for each chosen measurement | 66 | 62.1 | 3.64 | 1.248 | 35 | 37.1 | 2.97 | 0.961 |
| 68 | Data Collection & Measurements | e) describing the setting of the data collection as well as the dynamics (e.g. labor or real setting. participants were grouped into teams. worked collaboratively or individually. predefined tasks to be completed). | | - | - | - | 35 | 82.9 | 4.26 | 0.980 |
| 69 | Data Collection & Measurements | f) describing the role of the researchers in the evaluation (e.g. supporting role. silent observer). | | - | - | - | 35 | 77.1 | 4.09 | 1.173 |
| | Sampling | Describe who participated by… | | | | | | | | |

| | | | | | | | | | | |
|---|---|---|---|---|---|---|---|---|---|---|
| 36 | Sampling | a) describing any eligibility criteria for participation. | 66 | 90.9 | 4.53 | 0.845 | - | pc | - | - |
| 37 | Sampling | b) describing the role and expertise of the participants (e.g. academics. developers. potential end-users. other affected people). | 66 | 86.4 | 4.50 | 0.809 | - | pc | - | - |
| 38 | Sampling | c) explaining how it is ensured that the people who were involved were the people who fit the research question (e.g. stakeholder analysis. different digital health literacy levels). | 66 | 74.2 | 4.11 | 0.930 | 35 | 85.7 | 4.29 | 0.926 |
| | Recruitment & Engagement Strategies | Describe in detail how participants were recruited, and which strategies were used to engage them by | | | | | | | | |
| 39 | Recruitment & Engagement Strategies | a) explaining how participants were selected. e.g. purposive. convenience. consecutive. snowball | 66 | 84.8 | 4.42 | 0.878 | - | pc | - | - |
| 40 | Recruitment & Engagement Strategies | b) outlining how participants were approached. e.g. face-to-face. tele-phone. e-mail | 66 | 75.8 | 4.02 | 1.102 | - | pc | - | - |
| 41 | Recruitment & Engagement Strategies | c) describing how. how often. and where the study was announced or ad-vertised | 66 | 60.6 | 3.52 | 1.243 | 35 | 31.4 | 3.09 | 0.981 |
| 42 | Recruitment & Engagement Strategies | d) reporting relevant dates. periods of recruitment and follow-ups | 66 | 57.6 | 3.67 | 1.194 | 35 | 54.3 | 3.37 | 1.165 |
| 44 | Recruitment & Engagement Strategies | e) Reporting if there were any incentives offered (e.g., monetary or non-monetary incentives such as an offer to provide the survey results) | 66 | 77.3 | 4.11 | 0.994 | - | pc | - | - |
| 13 | Recruitment & Engagement Strategies | f) describing any approaches of engaging participants (e.g. strong and bal-anced advisory board with clinical. patients. technical team members able to lead the product design and development. co-creation work-shops). Describe formative research and/or content and/or usability test-ing with target group(s) clearly identified. as appropriate. | 66 | 87.9 | | | - | pc | - | - |
| 70 | Recruitment & Engagement Strategies | g) adding to the appendix a description of any material used to support the engagement in the evaluation (e.g. slides to explain concepts, templates to be filled out by participants). | | - | - | - | 35 | 60.0 | 3.49 | 0.951 |
| 75 | Recruitment & Engagement Strategies | h) providing a statement on the science communication strategy. | | - | - | - | | - | - | - |
| Results | | | | | | | | | | |
| 45 | Main Evaluation Results | Report the most relevant results for the selected and previously described variables of interest and summarize them in the most appropriate total for each methodological step. If applicable. report any conceptual or theoret-ical developments that have emerged regarding the participation. If appli-cable. include a relevance or significance analysis of the findings. | 66 | 90.9 | 4.67 | 0.847 | - | pc | - | - |
| 46 | Participants | a) Describe detailed numbers and expertise of participants involved at each stage of the study (e.g. numbers potentially eligible, examined for eli-gibility, confirmed eligible, included in the study, completing follow-up, and analyzed). | 66 | 81.8 | 4.32 | 0.914 | - | pc | - | - |

| | | | | | | | | | | |
|---|---|---|---|---|---|---|---|---|---|---|
| 43 | Participants | b) Describe the number of drop-outs and their associated context (e.g. how they were responded to, if applicable). | 66 | 57.6 | 3.58 | 1.164 | 35 | 60.0 | 3.74 | 1.146 |
| 49 | Participant checking | Report if participants provide feedback on the findings. | 66 | 78.8 | 4.20 | 0.898 | - | pc | - | - |
| 71 | Selective Reporting | To avoid selective reporting and risk of bias. report if any findings (e.g. negative findings) or unexpected results are not detailed described. Explain why. | | - | - | - | 35 | 65.7 | 3.69 | 1.301 |
| 73 | Science Communication Strategy | Describe the accessibility and availability of the results to the general public (e.g. open access, open source). | | | | | | | | |
| 48 | Impacts/Effects of participation | If applicable. report shortly the measured impact as well as barriers and facilitators of participation | 66 | 66.7 | 3.68 | 1.349 | 35 | 65.7 | 3.83 | 1.200 |
| Discussion | | | | | | | | | | |
| 50 | Key Findings | Report the key findings of the study and refer to the initial aim of the study. State. what is the (overall) assessment of the intervention. | 66 | 92.4 | 4.56 | 0.825 | - | pc | - | - |
| 54 | Results in relation to other studies | Place your results within the current state of research. State if it supports existing work. expands or challenges other findings. discuss possible reasons | 66 | 86.4 | 4.35 | 0.794 | - | pc | - | - |
| 53 | Implications | Explain whether now or in the future the findings of this study are used to develop or improve a DHI. Highlight the potential influence on digital health design and policy. | 66 | 89.4 | 4.42 | 0.786 | - | pc | - | - |
| 52 | Generalizability | Discuss to what extent the study results could be generalized for other DHIs. populations or organizations | 66 | 83.3 | 4.17 | 0.938 | - | pc | - | - |
| 55 | Lessons Learned | Describe any lessons learned from the participatory evaluation of DHI that could be used to improve future evaluations and outcomes. Reflect on the things that went well and those that did not (e.g. success factors. challenges or budget considerations) | 66 | 87.9 | 4.41 | 0.894 | - | pc | - | - |
| 51 | Limitations | Acknowledge the limitations of the study. including methodological. ethical and equity considerations. Address potential impacts on patients. policy or practice. and highlight challenges or biases in the participatory approach. | 66 | 90.9 | 4.59 | 0.859 | - | pc | - | - |
| 56 | Future Work | Describe your plans or give recommendations for future research. Refer to the limitations you mentioned. give concrete ideas how further areas could be examined or how your results could be integrated in practical implementations | 66 | 83.3 | 4.30 | 0.859 | - | pc | - | - |
| | Reflecting participation | Discuss the process and the results of the participation by ... | | | | | | | | |
| 57 | Reflecting participation | a) commenting. if it was possible to carry out the participation as planned. Reflect on what are the facilitators and barriers for participants to participate in the research and design process. | 66 | 74.2 | 3.98 | 0.903 | 35 | 82.9 | 4.14 | 0.879 |
| 58 | Reflecting participation | b) describing how participation influenced the study overall. Report (intended or unintended) positive and negative impacts that participation has had on the research. the participants involved (including patients and researchers). and wider impacts. Report on any difference the involvement made to the approach or findings of the study. | 66 | 81.8 | 4.21 | 0.903 | - | pc | - | - |

| | | | | | | | | | | | |
|---|---|---|---|---|---|---|---|---|---|---|---|
| 59 | Reflecting partici-pation | c) reporting the influence of any process or contextual factors. that ena-bled or hindered the impact of participation. Comment on how process factors and contextual factors influenced participation in the study | 66 | 78.8 | 4.09 | 0.872 | - | pc | - | - |
| 60 | Reflecting partici-pation | d) If applicable. describing on the rigor of the method used to capture or measure the impact as well as barriers and facilitators of participation. | 66 | 57.6 | 3.62 | 1.237 | 35 | 57.1 | 3.74 | 0.919 |
| 61 | Reflecting partici-pation | d) mentioning barriers or facilitators to the adoption of the intervention among study participants. Relates to individual-level structural. economic and social barriers or facilitators to access such as affordability. and other factors that may limit a user's ability to adopt the intervention. | 66 | 75.8 | 4.05 | 1.195 | - | pc | - | - |
| Others | | | | | | | | | | |
| 62 | Funding | Describe sources of funding and other support | 66 | 81.8 | 4.27 | 1.060 | - | pc | - | - |
| 63 | Conflict of inter-ests | In addition to the usual declaration of interests (financial or otherwise). also state the "relation of the study team towards the system being evalu-ated". i.e. state if the authors/evaluators are distinct from or identical with the developers/sponsors of the intervention. | 66 | 86.4 | 4.45 | 1.040 | - | pc | - | - |
| 64 | Contributions | Share a detailed description of the contributions of the authors to the published work (e.g., Using CRediT). Report if participating citizens or pa-tients did co-author and report contributions of team members that are not authors. | 66 | 80.3 | 4.12 | 1.17 | - | pc | - | - |
| 72 | Commercializa-tion | Describe if the DHI is going to be commercialized by a company (e.g., startup. spin-off). | | - | - | - | 35 | 54.3 | 3.46 | 1.502 |

Supplementary Information S3: Detailed Results 1. Round

| OID | Item Title | Item- full text | 1- Very unim-portant | 1 (%) | 2 | 2 (%) | 3 | 3 (%) | 4 | 4 (%) | 5- Very im-portant | 5 (%) | Can-not rate this item | Can-not rate this item (%) | CR (%) | Mean | SD | Consequences from qualitative comments |
|---|---|---|---|---|---|---|---|---|---|---|---|---|---|---|---|---|---|---|
| Abstract | | | | | | | | | | | | | | | | | | |
| 1 | Aim | State the aim of the participatory evaluation | 3 | 4.5 | 0 | 0.0 | 2 | 3.0 | 8 | 12.1 | 52 | 78.8 | 1 | 1.5 | 90.9 | 4.56 | 1.083 | |
| 2 | Method | Describe the key methods for evaluation and participation, such as including evaluated criteria & indicators. | 2 | 3.0 | 4 | 6.1 | 3 | 4.5 | 10 | 15.2 | 47 | 71.2 | 0 | 0.0 | 86.4 | 4.45 | 1.040 | Added augmentations based on comments 1c, 1k, 1o, 1r, 1v, 1w, 2d, Supplementary Information S4 |
| 3 | Results | Report the key findings and their significance | 3 | 4.5 | 1 | 1.5 | 5 | 7.6 | 13 | 19.7 | 44 | 66.7 | 0 | 0.0 | 86.4 | 4.42 | 1.024 | Reworded item based on comment 2c, Supplementary Information S4 |
| 4 | Conclusion | Summarize the main conclusions of the study and potential implications | 2 | 3.0 | 1 | 1.5 | 6 | 9.1 | 13 | 19.7 | 44 | 66.7 | 0 | 0.0 | 86.4 | 4.45 | 0.948 | |
| 5 | Keywords | List relevant keywords that represent the core topics of the study | 0 | 0.0 | 15 | 22.7 | 12 | 18.2 | 19 | 28.8 | 22 | 33.3 | 0 | 0.0 | 62.1 | 3.67 | 1.141 | |
| Background & Foundation | | | | | | | | | | | | | | | | | | |
| 6 | Scientific Background | Describe the scientific background of the study and indicate which research was carried out in advance and which databases and terms were used to find information. | 1 | 1.5 | 3 | 4.5 | 6 | 9.1 | 22 | 33.3 | 34 | 51.5 | 0 | 0.0 | 84.8 | 4.29 | 0.924 | |
| 7 | Study Context | Describe the context of the study and the addressed problem. Describe whether the study is part of a larger research, development or implementation project. Describe the history/development process of the application and previous formative evaluations (e.g. focus groups, usability testing). | 2 | 3.0 | 0 | 0.0 | 7 | 10.6 | 22 | 33.3 | 35 | 53.0 | 0 | 0.0 | 86.4 | 4.33 | 0.900 | |

| | | | | | | | | | | | | | | | | |
|---|---|---|---|---|---|---|---|---|---|---|---|---|---|---|---|---|
| | | If possible, it should be mentioned what influence the findings of the study have. Mention from which stakeholder viewpoint (if any) the study is performed. | | | | | | | | | | | | | | |
| 8 | Rationale | Describe the motivation and specific reasons for the study (scientific interest, justification for expenditure, insight into problems, addressing open research questions). | 1 | 1.5 | 2 | 3.0 | 5 | 7.6 | 17 | 25.8 | 41 | 62.1 | 0 | 0.0 | 87.9 | 4.44 | 0.879 | Reworded item based on comment 5h, Supplementary Information S4 |
| 9 | Ethical Vote & Considerations | Please mention the ethical vote for the survey and where it was obtained. Add information about the consideration of the possibility of harm as well as the assessment of the likelihood of risks as well as adverse outcomes. | 4 | 6.1 | 6 | 9.1 | 5 | 7.6 | 15 | 22.7 | 36 | 54.5 | 0 | 0.0 | 77.3 | 4.11 | 1.242 | |
| 10 | Participation Concept | Report the definition of participation used in the study and the belonging concept (e.g., PPI, PHR..). Describe why this definition fits your study. | 1 | 1.5 | 1 | 1.5 | 10 | 15.2 | 17 | 25.8 | 36 | 54.5 | 1 | 1.5 | 80.3 | 4.26 | 1.042 | |
| 11 | Participation Concept | Describe what methodological orientation was stated to underpin the study, e.g. grounded theory, discourse analysis, ethnography, phenomenology, content analysis. | 2 | 3.0 | 3 | 4.5 | 13 | 19.7 | 15 | 22.7 | 33 | 50.0 | 0 | 0.0 | 72.7 | 4.12 | 1.074 | Relocate into method, based on comment e44, Supplementary Information S4 |
| | Participation Concept | Report the level or nature of participation used at various stages of the study by | | | | | | | | | | | | | | | |
| 12 | Participation Concept | a) referring to established stage models of participation like Wright et al. | 7 | 10.6 | 4 | 6.1 | 17 | 25.8 | 19 | 28.8 | 19 | 28.8 | 0 | 0.0 | 57.6 | 3.48 | 1.501 | Merged with item 26, based on comment 6c, Supplementary Information S4 |
| 13 | Participation regarding DHI development | b) describing any approaches to engage patients or service recipients, the general public, communities or stakeholders | 1 | 1.5 | 0 | 0.0 | 7 | 10.6 | 23 | 34.8 | 35 | 53.0 | 0 | 0.0 | 87.9 | 4.38 | 0.799 | Relocate into method, based on comment e44, |

| | | | | | | | | | | | | | | | | | | |
|---|---|---|---|---|---|---|---|---|---|---|---|---|---|---|---|---|---|---|
| | | in the DHI development (e.g., strong and balanced advisory board with clinical/patients/technical team members able to lead the product design and development, co-creation workshops with patients). Describe formative research and/or content and/or usability testing with target group(s) clearly identified, as appropriate. | | | | | | | | | | | | | | | | Supplementary Information S4 |
| 14 | Participation concept for vulnerable groups | c) outlining how it was ensured that vulnerable groups were adequately considered and represented in the research. | 1 | 1.5 | 3 | 4.5 | 7 | 10.6 | 20 | 30.3 | 34 | 51.5 | 1 | 1.5 | 81.8 | 4.21 | 1.074 | Merged with item 31, based on comment 6e, Supplementary Information S4 |
| | DHI Description | Describe the type of the DHI that is the object of the study by...<br>(If there is one or more comparators all of the following points also have to be addressed) | | | | | | | | | | | | | | | | |
| 15 | DHI Description- Classification System | a) referring to established classification systems (e.g. those provided by the WHO/NICE). | 0 | 0.0 | 2 | 3.0 | 16 | 24.2 | 18 | 27.3 | 21 | 31.8 | 9 | 13.6 | 59.1 | 3.47 | 1.619 | Added augmentation based on comment 1j and 4s, Supplementary Information S4 |
| 16 | DHI Description- Key Components | b) listing the characteristics and key components of the DHI. Outline its primary functions as well as its user interface. Describe how the components interact with each other and which components impact on which predicted outcomes. | 0 | 0.0 | 2 | 3.0 | 9 | 13.6 | 12 | 18.2 | 41 | 62.1 | 2 | 3.0 | 80.3 | 4.30 | 1.136 | |
| 17 | DHI Description- Fit to target group | c) describing which target group the DHI addresses and why it is likely to reach this population as well as why the population will be likely to use it. Add information on the health domain it addresses, the specific medical issue it | 0 | 0.0 | 3 | 4.5 | 6 | 9.1 | 15 | 22.7 | 40 | 60.6 | 2 | 3.0 | 83.3 | 4.30 | 1.136 | |

| | | | | | | | | | | | | | | | | | | |
|---|---|---|---|---|---|---|---|---|---|---|---|---|---|---|---|---|---|---|
| | | targets and the intended use setting. | | | | | | | | | | | | | | | | |
| 18 | DHI Description-Access for vulnerable populations | d) outlining how access for vulnerable populations is secured (e.g., adjustment of text size, text to voice, colorblind color scheme adjuster, specificity to use by minors, offline features, left and right handed options). State how the DHI takes the user context into account and does not vary in quality because of personal characteristics such as dexterity, disabilities, movement disorders or vision problems etc. | 1 | 1.5 | 1 | 1.5 | 8 | 12.1 | 21 | 31.8 | 33 | 50.0 | 2 | 3.0 | 81.8 | 4.18 | 1.136 | Reworded based on comments 4b, 4e, 4f, 4g, 4h, 4q, Supplementary Information S4 |
| 19 | DHI Description-Updating and revisions | e) mentioning the date and/or version number of the DHI or describe whether the intervention underwent major changes during the evaluation process or whether the development and/or content was "frozen" during the trial. Describe dynamic components such as news feeds or changing content which may have an impact on the replicability of the intervention. | 0 | 0.0 | 4 | 6.1 | 12 | 18.2 | 23 | 34.8 | 23 | 34.8 | 4 | 6.1 | 69.7 | 3.80 | 1.315 | Reworded based on comments 1j and 4g, Supplementary Information S4 |
| 20 | DHI Description-Tailoring during use | f) describing whether the DHI is using ongoing feedback and appropriate call to action based on the user's state and activities (e.g. guidance based on user-centered information). Describe whether there are any strategies to support tailoring the DHI to participants over time. | 1 | 1.5 | 2 | 3.0 | 11 | 16.7 | 30 | 45.5 | 17 | 25.8 | 5 | 7.6 | 71.2 | 3.68 | 1.349 | Reworded based on comments 3e, 4g and 4i, Supplementary Information S4 |
| Method | | | | | | | | | | | | | | | | | | |
| 21 | Study design | Present key study design (participation & evaluation), including the mention of a possible pre-registration | 2 | 3.0 | 0 | 0.0 | 6 | 9.1 | 11 | 16.7 | 47 | 71.2 | 0 | 0.0 | 87.9 | 4.53 | 0.898 | |

| | | | | | | | | | | | | | | | | | | |
|---|---|---|---|---|---|---|---|---|---|---|---|---|---|---|---|---|---|---|
| 22 | Study flow | Visualize the research process for example by using the CONSORT flow chart | 1 | 1.5 | 6 | 9.1 | 9 | 13.6 | 22 | 33.3 | 25 | 37.9 | 3 | 4.5 | 71.2 | 3.83 | 1.319 | Added augmentation based on comment 6a, Supplementary Information S4 |
| 23 | Informed consent | Describe the informed consent process by where the participants were told the length of time of the survey, which data were stored and where and for how long, who the investigator was, and what the purpose of the study is. Add information about the possibilities of drop-out. | 3 | 4.5 | 7 | 10.6 | 7 | 10.6 | 19 | 28.8 | 30 | 45.5 | 0 | 0.0 | 74.2 | 4.00 | 1.190 | Reworded based on comments 6b, 6i, 6n, and 8f, Supplementary Information S4 |
| 24 | Impacts/Effects of participation | If applicable, report the methods used to explore the impact of participation in the study. | 0 | 0.0 | 4 | 6.1 | 15 | 22.7 | 18 | 27.3 | 27 | 40.9 | 2 | 3.0 | 68.2 | 3.94 | 1.175 | Added augmentation based on comment 10e, Supplementary Information S4 |
| | Participatory Evaluation Method | Explain the participatory evaluation methods used in the study by | | | | | | | | | | | | | | | | |
| 25 | Participatory Evaluation Method | a) explaining the choosen participatory methods used in the evaluation, such as co-design workshops, user testing, or participatory data analysis and defining the used evaluation criteria including their underlying indicators | 0 | 0.0 | 3 | 4.5 | 0 | 0.0 | 15 | 22.7 | 48 | 72.7 | 0 | 0.0 | 95.5 | 3.64 | 0.715 | Reworded based on comment 5f, Supplementary Information S4 |
| 26 | Participatory Evaluation Method | b) describing the level or nature of participation used at various stages of the study. Refer to established stage models of participation (eg., Wright et al.) | 1 | 1.5 | 1 | 1.5 | 12 | 18.2 | 24 | 36.4 | 26 | 39.4 | 2 | 3.0 | 75.8 | 4.02 | 1.130 | Relocate into method, based on comments 1j, 4ee, Supplementary Information S4.<br>Merged with item 12, bas ed on comment 6c.<br>Reworded based on comment 1i and 4a. |
| 27 | Participatory Evaluation Method | c) describe any approaches to participate or engage patients or service recipients, the general public, | 0 | 0.0 | 3 | 4.5 | 8 | 12.1 | 27 | 40.9 | 27 | 40.9 | 1 | 1.5 | 81.8 | 4.14 | 0.975 | Reworded based on comments 4, and 5l, Supplementary Information S4 |

| | | | | | | | | | | | | | | | | | |
|---|---|---|---|---|---|---|---|---|---|---|---|---|---|---|---|---|---|
| | | communities, or stakeholders (such as clinicians or payers) in the design of the study. | | | | | | | | | | | | | | | |
| 28 | Participatory Evaluation Method | d) describing the reasons behind the chosen evaluation methods | 2 | 3.0 | 1 | 1.5 | 11 | 16.7 | 19 | 28.8 | 33 | 50.0 | 0 | 0.0 | 78.8 | 4.21 | 0.985 | Added augmentation based on comment 5n, Supplementary Information S4 |
| 29 | Participatory Evaluation Method | e) referring to existing frameworks (e.g. WHO, NICE, EXPH, the Swiss Evaluation framework by Kowatsch et al, the approach of Murray et al. or others) | 2 | 3.0 | 1 | 1.5 | 16 | 24.2 | 22 | 33.3 | 19 | 28.8 | 6 | 9.1 | 62.1 | 3.56 | 1.469 | Reworded based on comments for mentioning more references, see 13a- 13x, Supplementary Information S4 |
| 30 | Participatory Evaluation Method | f) explaining the reasons for the exclusion briefly, if established methods are deemed inappropriate or inapplicable for the specific context of the DHI being evaluated. | 1 | 1.5 | 6 | 9.1 | 10 | 15.2 | 25 | 37.9 | 22 | 33.3 | 2 | 3.0 | 71.2 | 3.83 | 1.210 | Reworded based on comments 6d, and 6q, Supplementary Information S4 |
| 31 | Participatory Evaluation Method | g) outline how it was ensured that vulnerable groups were adequately considered and represented in the participatory evaluation. | 2 | 3.0 | 3 | 4.5 | 9 | 13.6 | 24 | 36.4 | 27 | 40.9 | 1 | 1.5 | 77.3 | 4.03 | 1.123 | Relocate into method, based on comment e44, Supplementary Information S4.<br>Merged with item 14, based on comment 6e.<br>Added augmentation based on item 4b. |
| | Data Collection & Measurements | Provide details on the data collection methods used for each variable of interest, by | | | | | | | | | | | | | | | |
| 32 | Data Collection & Measurements | a) describing all relevant aspects of applied data collection methods, e.g. questionnaire, interview, focus groups, observation, task-/scenario-completion, log file analysis | 0 | 0.0 | 1 | 1.5 | 6 | 9.1 | 10 | 15.2 | 49 | 74.2 | 0 | 0.0 | 89.4 | 4.62 | 0.718 | |
| 33 | Data Collection & Measurements | b) explaining which roles established measurements play in the study. If established measurements can't be used, explain why. | 0 | 0.0 | 2 | 3.0 | 13 | 19.7 | 25 | 37.9 | 22 | 33.3 | 4 | 6.1 | 71.2 | 3.83 | 1.272 | |

| | | | | | | | | | | | | | | | | | |
|---|---|---|---|---|---|---|---|---|---|---|---|---|---|---|---|---|---|
| 34 | Data Collection & Measurements | c) If newly designed measurements are used, describe in detail all aspects of instrument development and testing (e.g., validity, reliability, feasibility, acceptability, responsiveness, interpretability, appropriateness, precision). If applicable, add supplementary material to appendices. | 0 | 0.0 | 3 | 4.5 | 10 | 15.2 | 21 | 31.8 | 31 | 47.0 | 1 | 1.5 | 78.8 | 4.17 | 1.017 | Reworded based on comment 6g, Supplementary Information S4 |
| 35 | Data Collection & Measurements | d) describing the comparability for each chosen measurement | 1 | 1.5 | 5 | 7.6 | 16 | 24.2 | 24 | 36.4 | 17 | 25.8 | 3 | 4.5 | 62.1 | 3.64 | 1.248 | |
| | Sampling | Describe who participated by… | | | | | | | | | | | | | | | | |
| 36 | Sampling | a) describing any eligibility criteria for participation | 1 | 1.5 | 2 | 3.0 | 3 | 4.5 | 15 | 22.7 | 45 | 68.2 | 0 | 0.0 | 90.9 | 4.53 | 0.845 | |
| 37 | Sampling | b) describing the role and expertise of the participants (academics, developers, potential end-users) and stakeholders | 0 | 0.0 | 2 | 3.0 | 7 | 10.6 | 13 | 19.7 | 44 | 66.7 | 0 | 0.0 | 86.4 | 4.50 | 0.809 | Reworded for clarity, based on Authors' team |
| 38 | Sampling | c) explaining how it is ensured that the people who were involved were the right people (e.g., stakeholder analysis, different digital health literacy levels) | 0 | 0.0 | 4 | 6.1 | 13 | 19.7 | 21 | 31.8 | 28 | 42.4 | 0 | 0.0 | 74.2 | 4.11 | 0.930 | Reworded based on comment 5l, and 6h, Supplementary Information S4 |
| | Recruitment & Engagement Strategies | Describe in detail how participants were recruited, and which strategies were used to engage them by | | | | | | | | | | | | | | | | |
| 39 | Recruitment & Engagement Strategies | a) explaining how participants were selected, e.g. purposive, convenience, consecutive, snowball | 1 | 1.5 | 1 | 1.5 | 8 | 12.1 | 15 | 22.7 | 41 | 62.1 | 0 | 0.0 | 84.8 | 4.42 | 0.878 | |
| 40 | Recruitment & Engagement Strategies | b) outlining how participants were approached, e.g. face-to-face, telephone, e-mail | 3 | 4.5 | 4 | 6.1 | 9 | 13.6 | 23 | 34.8 | 27 | 40.9 | 0 | 0.0 | 75.8 | 4.02 | 1.102 | |
| 41 | Recruitment & Engagement Strategies | c) describing how, how often, and where the survey was announced or advertised | 6 | 9.1 | 9 | 13.6 | 11 | 16.7 | 25 | 37.9 | 15 | 22.7 | 0 | 0.0 | 60.6 | 3.52 | 1.243 | Reworded based on comment 5l, Supplementary Information S4 |

| | | | | | | | | | | | | | | | | | | |
|---|---|---|---|---|---|---|---|---|---|---|---|---|---|---|---|---|---|---|
| 42 | Recruitment & Engagement Strategies | d) reporting relevant dates, periods of recruitment and follow-ups | 2 | 3.0 | 7 | 10.6 | 18 | 27.3 | 18 | 27.3 | 20 | 30.3 | 1 | 1.5 | 57.6 | 3.67 | 1.194 | |
| 43 | Recruitment & Engagement Strategies | e) describing how attempts are made to counteract a drop-out | 4 | 6.1 | 8 | 12.1 | 16 | 24.2 | 22 | 33.3 | 16 | 24.2 | 0 | 0.0 | 57.6 | 3.58 | 1.164 | Reworded based on comments 5l, and 6j, Supplementary Information S4 |
| 44 | Recruitment & Engagement Strategies | f) Reporting if there were any incentives offered (e.g., monetary or non-monetary incentives such as an offer to provide the survey results) | 2 | 3.0 | 2 | 3.0 | 11 | 16.7 | 23 | 34.8 | 28 | 42.4 | 0 | 0.0 | 77.3 | 4.11 | 0.994 | |
| Results | | | | | | | | | | | | | | | | | | |
| 45 | Main Evaluation Results | Report the most relevant results for the selected and previously described variables and summarize them in the most appropriate total. If applicable, report any conceptual or theoretical developments that have emerged regarding the participation. | 2 | 3.0 | 0 | 0.0 | 4 | 6.1 | 6 | 9.1 | 54 | 81.8 | 0 | 0.0 | 90.9 | 4.67 | 0.847 | Added augmentation based on comments 7g, and 8a, Supplementary Information S4 |
| 46 | Participants | Describe detailed number and expertise of humans involved at each stage of study - e.g., numbers potentially eligible, examined for eligibility, confirmed eligible, included in the study, completing follow-up, and analyzed. As well, add a type of assistance offered, the timing and frequency of the support, how it is initiated, and the medium by which the assistance is delivered. | 0 | 0.0 | 4 | 6.1 | 8 | 12.1 | 17 | 25.8 | 37 | 56.1 | 0 | 0.0 | 81.8 | 4.32 | 0.914 | Reworded based on comment 8g, Supplementary Information S4 |
| 47 | Impacts/Effects of participation | Report (intended or unintended) positive and negative impacts that participation has had on the research, the individuals involved (including patients and researchers), and wider impacts. Report on any difference patient/service | 2 | 3.0 | 1 | 1.5 | 8 | 12.1 | 22 | 33.3 | 32 | 48.5 | 1 | 1.5 | 81.8 | 4.18 | 1.080 | Relocated based on comment 9g, Supplementary Information S4. Merged with item58 based on comment 8h, Supplementary Information S4. |

| | | | | | | | | | | | | | | | | | |
|---|---|---|---|---|---|---|---|---|---|---|---|---|---|---|---|---|---|
| | | recipient, general public, community, or stakeholder involvement made to the approach or findings of the study. | | | | | | | | | | | | | | | |
| 48 | Impacts/Effects of participation | If applicable, report the rigor of the method used to capture or measure the impact of participation. | 1 | 1.5 | 5 | 7.6 | 12 | 18.2 | 24 | 36.4 | 20 | 30.3 | 4 | 6.1 | 66.7 | 3.68 | 1.349 | Reworded based on comment 8d, Supplementary Information S4. |
| 49 | Participant checking | Report if participants provide feedback on the findings | 1 | 1.5 | 1 | 1.5 | 12 | 18.2 | 22 | 33.3 | 30 | 45.5 | 0 | 0.0 | 78.8 | 4.20 | 0.898 | |
| Discussion | | | | | | | | | | | | | | | | | | |
| 50 | Key Findings | Report the key findings of the study and refer to the initial aim of the study. State, what is the (overall) assessment of the intervention | 1 | 1.5 | 2 | 3.0 | 2 | 3.0 | 15 | 22.7 | 46 | 69.7 | 0 | 0.0 | 92.4 | 4.56 | 0.825 | Reworded based on comment 10l, Supplementary Information S4. |
| 51 | Limitations | Acknowledge the limitations of the study. Discuss restrictions of your research design or methodological choices. Take into account whether there are ethical, or equity considerations not captured and if so, if there are any impacts on patients, policy, or practice. | 2 | 3.0 | 0 | 0.0 | 4 | 6.1 | 11 | 16.7 | 49 | 74.2 | 0 | 0.0 | 90.9 | 4.59 | 0.859 | |
| 52 | Generalizability | Discuss to what extent the study results could be generalized for other DHIs, populations or organizations | 1 | 1.5 | 4 | 6.1 | 6 | 9.1 | 27 | 40.9 | 28 | 42.4 | 0 | 0.0 | 83.3 | 4.17 | 0.938 | |
| 53 | Implications | Explain whether now or in the future the findings of this study are used to develop or improve a DHI. If applicable describe how users can gain access to the DHI | 1 | 1.5 | 0 | 0.0 | 6 | 9.1 | 22 | 33.3 | 37 | 56.1 | 0 | 0.0 | 89.4 | 4.42 | 0.786 | Reworded based on comment 10l, Supplementary Information S4. |
| 54 | Results in relation to other studies | Place your results within the current state of research. State if it supports existing work, expands or challenges other findings, discuss possible reasons | 0 | 0.0 | 2 | 3.0 | 7 | 10.6 | 23 | 34.8 | 34 | 51.5 | 0 | 0.0 | 86.4 | 4.35 | 0.794 | |
| 55 | Lessons Learned | Describe any lessons learned from the participatory | 2 | 3.0 | 0 | 0.0 | 6 | 9.1 | 19 | 28.8 | 39 | 59.1 | 0 | 0.0 | 87.9 | 4.41 | 0.894 | |

| | | | | | | | | | | | | | | | | | | |
|---|---|---|---|---|---|---|---|---|---|---|---|---|---|---|---|---|---|---|
| | | evaluation of DHI that could be used to improve future evaluations and outcomes. Reflect on the things that went well and those that did not (e.g. success factors, challenges or budget considerations) | | | | | | | | | | | | | | | | |
| 56 | Future Work | Describe your plans or give recommendations for future research. Refer to the limitations you mentioned, give concrete ideas how further areas could be examined or how your results could be integrated in practical implementations | 0 | 0.0 | 3 | 4.5 | 8 | 12.1 | 21 | 31.8 | 34 | 51.5 | 0 | 0.0 | 83.3 | 4.30 | 0.859 | |
| | Reflecting participation | Discuss the process and the results of the participation by | | | | | | | | | | | | | | | | |
| 57 | Reflecting participation | a) commenting, if it was possible to carry out the participation as planned | 0 | 0.0 | 5 | 7.6 | 12 | 18.2 | 28 | 42.4 | 21 | 31.8 | 0 | 0.0 | 74.2 | 3.98 | 0.903 | Reworded based on comments 9j, 10b, and 10l, Supplementary Information S4. |
| 58 | Reflecting participation | b) commenting on how participation influenced the study overall. Describe the positive and negative effects | 0 | 0.0 | 3 | 4.5 | 12 | 18.2 | 19 | 28.8 | 32 | 48.5 | 0 | 0.0 | 77.3 | 4.21 | 0.903 | |
| 59 | Reflecting participation | c) reporting the influence of any process or contextual factors, that enabled or hindered the impact of participation. Comment on how process factors and contextual factors influenced participation in the study | 0 | 0.0 | 4 | 6.1 | 10 | 15.2 | 28 | 42.4 | 24 | 36.4 | 0 | 0.0 | 78.8 | 4.09 | 0.872 | |
| 60 | Reflecting participation | d) If applicable, commenting on how well participation impact was evaluated or measured in the study | 0 | 0.0 | 6 | 9.1 | 19 | 28.8 | 20 | 30.3 | 18 | 27.3 | 3 | 4.5 | 57.6 | 3.62 | 1.237 | Reworded based on comments 10l, and 10l Supplementary Information S4. |
| 61 | Reflecting participation | e) mentioning barriers or facilitators to the adoption of the intervention among study participants. Relates to individual-level structural, | 1 | 1.5 | 3 | 4.5 | 10 | 15.2 | 20 | 30.3 | 30 | 45.5 | 2 | 3.0 | 75.8 | 4.05 | 1.195 | |

| | | | | | | | | | | | | | | | | | | |
|---|---|---|---|---|---|---|---|---|---|---|---|---|---|---|---|---|---|---|
| | | economic and social barriers or facilitators to access such as affordability, and other factors that may limit a user's ability to adopt the intervention | | | | | | | | | | | | | | | | |
| 62 | Funding | Describe sources of funding and other support | 2 | 3.0 | 4 | 6.1 | 6 | 9.1 | 16 | 24.2 | 38 | 57.6 | 0 | 0.0 | 81.8 | 4.27 | 1.060 | |
| 63 | Conflict of interests | In addition to the usual declaration of interests (financial or otherwise), also state the "relation of the study team towards the system being evaluated", i.e., state if the authors/evaluators are distinct from or identical with the developers/sponsors of the intervention. | 2 | 3.0 | 4 | 6.1 | 3 | 4.5 | 10 | 15.2 | 47 | 71.2 | 0 | 0.0 | 86.4 | 4.45 | 1.040 | |
| 64 | Contributions | Share a detailed description of the contributions of the authors to the published work (e.g., Using CRediT). | 4 | 6.1 | 4 | 6.1 | 5 | 7.6 | 20 | 30.3 | 33 | 50.0 | 0 | 0.0 | 80.3 | 4.12 | 1.17 | Added augmentation based on comment 11a, and 12d, Supplementary Information S4. |
| Certainty | | | | | | | | | | | | | | | | | | |
| | Certainty Abstract | | 0 | 0.0 | 2 | 3.0 | 4 | 6.1 | 22 | 33.3 | 38 | 57.6 | 0 | 0.0 | 90.9 | 4.45 | 0.748 | |
| | Certainty Background | | 0 | 0.0 | 1 | 1.5 | 8 | 12.1 | 39 | 59.1 | 18 | 27.3 | 0 | 0.0 | 86.4 | 4.12 | 0.668 | |
| | Certainty Method | | 0 | 0.0 | 0 | 0.0 | 7 | 10.6 | 31 | 47.0 | 28 | 42.4 | 0 | 0.0 | 89.4 | 4.32 | 0.660 | |
| | Certainty Results | | 0 | 0.0 | 0 | 0.0 | 6 | 9.1 | 29 | 43.9 | 31 | 47.0 | 0 | 0.0 | 90.9 | 4.38 | 0.651 | |
| | Certainty Discussion | | 0 | 0.0 | 1 | 1.5 | 10 | 15.2 | 26 | 39.4 | 29 | 43.9 | 0 | 0.0 | 83.3 | 4.26 | 0.771 | |
| | Certainty Others | | 0 | 0.0 | 0 | 0.0 | 5 | 7.6 | 20 | 30.3 | 41 | 62.1 | 0 | 0.0 | 92.4 | 4.55 | 0.637 | |

## Supplementary Information S4: Qualitative Results 1. Round

| Origin free-text field | Comment | Category | Effect of comment |
|---|---|---|---|
| 1. Comments regarding missing items in Abstract section | a. Short background of the studs | Revising scope | Added new item 65 |
| | b. 1.2 who participated in evaluation: number of patients, citizens, and some main characteristics | Revising existing content | Added augmentation into items 2 & 3 |
| | c. I'd suggest to also explicitly ask to report the key characteristics of the people involved in the participatory evaluation process (sample size, sociodemographic characteristics, role, and phase of the design and/or evaluation process in which they were involved). | Revising existing content | Added augmentation into item 2 |
| | d. na | Others | . |
| | e. no not really - i think it is good | General approval | . |
| | f. It should briefly describe the digital solution or intervention assessed and the target population | Revising existing content | Content is covered by items 15- 18 |
| | g. Short background | Revising scope | Added new item 65 |
| | h. Trial registration if appropriate; specify who was engaged in participatory approach; | Revising existing content | Content is covered by item 37 |
| | i. Regulatory pathway | Revising scope | Content is covered by various items |
| | j. Not essential, but some reference to any theoretical framework for characterizing the level of type of involvement may be helpful | Revising existing content | Added augmentation into various items, like 15, 19, 26 |
| | k. The target group of the study | Revising existing content | Added augmentations into item 2 |
| | l. Participants: Report who was involved in the participatory development. | Revising existing content | Content is covered by item 37 |
| | m. If the study is registered, the link to registration. | Revising scope | . |
| | n. I would not say it's missing, because it might go up in "METHOD", but to me it's also very important to indicate the sample/the groups of people, that evaluated the DHI | Revising existing content | Content is already covered by items 36- 38 |
| | o. "geographic place of data collection and age of the participants (especially if those are children) is important for me. Because not in all setting PE can be conducted." | Revising existing content | Added augmentation into item 2 |
| | p. No | General approval | |
| | q. 1.4 Conclusion could also contain potential limitations and implications of these limitations. | Revising existing content | . |
| | r. In the methods, it would be good to report on the Population intended to use the intervention and the population engaged in the design, development, implementation, and evaluation - including gender distribution of participants and any other relevant demographic information such as ethnicity, rural /urban, age, etc. As more data from digital health interventions is used in AI/ML activities- bias and the potential for bias should be made more transparent in reporting. | Revising existing content | Added augmentations into item 2. Other content is covered in Methods section |
| | s. No i don't think so - it looks good for me | General approval | |
| | t. collaboration | Revising scope | Too unspecific to revise scope or existing content. |
| | u. Background, including state-of-the-art in that specific field | Revising scope | Added new item 65 |
| | v. Maybe the people involved in the participatory evaluation (e.g. end users, experts, etc.) | Revising existing content | Added augmentations into item 2 |
| | w. who was involved as participants: patients, users, parents, professionals ? How representative is this sample? | Revising existing content | Added augmentations into item 2 |
| | x. n/a | Others | |

| | | | |
|---|---|---|---|
| | y. None | General approval | |
| | z. novelty | Revising scope | Too unspecific to revise scope or existing content. |
| | aa. no | General approval | |
| 2. Further comments regarding Abstract section | a. na | Others | |
| | b. no not really | Others | |
| | c. Careful with the word "significance" in 1.3. In a non-quantitative approach, as many participatory methods are designed, this might seem out of place. I assume you mean "relevance", which I then find slightly negligible in an abstract which has no discussion section. | Revising existing content | Reworded item 3 |
| | d. 1.2 In the methods section the degree of participation and methods for participation should be included, e.g. "only" interviews or focus groups or co-production workshops, etc. | | Added augmentations into item 2 |
| | e. Provide the abstract in different languages including simple language (Einfache Sprache). | Revising scope | . |
| | f. A rather general remark: why should an additional reporting guideline be necessary? Studies with digital medicinal products need to follow ISO 14155, mandatory publications need to follow e.g. the CONSORT statement or journal requirements. I don't see, how an additional guideline would improve comparability. Heterogeneity with regard to DiGAs also mainly results from different indications. | General criticism | Necessity of additional reporting guideline is outlined in Introduction based on existing literature. |
| | g. make it less text - it is too complicated. | Revising scope | Noted as general orientation for proper handling of the guideline. |
| | h. No other comment | Others | |
| | i. n/a | Others | |
| | j. There is an interesting Delphi study, may you share the study findings once it can be disseminated? | General approval | |
| | k. None | Others | |
| | l. no | Others | |
| | m. i dont like the way this survey has been set up- 1 is usually a very important category, 5 is least important. You may get misclassified results." | General criticism | . |
| 3. Comments regarding missing items in Background section | a. na | Others | |
| | b. No i think it is very good | General approval | |
| | c. It should be specifically described: - if any BCTs is used, the theoretical background for this BC Intervention | Revising existing content | Content is covered by item 16 |
| | d. It should be specifically described:- Whether a design framework was followed in the development process of DHI and the digital health solution | Revising scope | Added new item 67 |
| | e. It should be specifically described:- Whether the DHI or DH solution is personalized and the key component of the personalization (what components are implemented in the DH solution)" | Revising existing content | Added augmentations into item 20 |
| | f. "- There is no item on theory or frameworks in a chapter to be called ""theoretical. At least one item concerning the reporting of underlying theories or frameworks (on implementation, user acceptance etc.) needs to be added! | Revising existing content | Added augmentation to topic of item 10 |
| | g. - Composition of the DHI development" | Revising scope | Added new item 67 |
| | h. describing which organisations should adopt the DHI, which resources are important for implementation in routine processes and maintenance | Revising scope | Added new item 66 |
| | i. Report potential unintended side effects of the digital health innovation. | Revising existing content | Content is covered by item 15- 20 |
| | j. If behavioral intervention is part of the DHI, the theoretical background should also address behavioral change frameworks used. | Revising existing content | Content is covered by item 15- 21 |
| | k. No | General approval | |

| | | | |
|---|---|---|---|
| | l. no but also soooo much text - we dont need that | Revising scope | Noted as general orientation for proper handling of the guideline. |
| | m. Tools used for evaluation, e.g. usability questionnaires, and how they were developed, adapted and evaluated, and whether they are appropriate for the different participant groups | Revising existing content | Content is covered by item 32 |
| | n. The use of other models and the fact that it should be clear which kind of population was examined (adults, children ...) | | Content is covered by items 36- 38 |
| | o. no | General approval | |
| | p. "What is the mechanism of action targeted, do we know how it is supposed to work | Revising existing content | Content is covered by item 16 |
| | q. What is the level of evidence of efficacy (e.g. exposure therapy or deep pressure) ?" | Revising existing content | Content is covered by item 16 |
| | r. n/a | Others | |
| | s. I think for item 2.7.2, the researchers should elaborate on how the co-design workshops were set up. Because there are so many different methods and tools to run the co-design workshops. In the discussion section: THe researchers should also reflect on the design process, what design methods and tools helped and what did not help; It would help future researchers to identify the effective methods and tools to use in the future. | Revising existing content | Content is covered by items 25, 26, 57, 58 59, 61 |
| | t. It would be good to acknowledge the disciplinary background and therefore epistemologies and sometimes ontologies that underpin the approach that has been used in developing the DHI. | Revising scope | Added new item 66 |
| | u. None | General approval | |
| | v. none | General approval | |
| 4. Further comments regarding Background section | a. No fan of stage models, as higher level is frequently considered better, in my opinion the level should fit the purpose and aim of participation and in some cases it is about resources that are lacking, this is why an explanation should be requested for the chosen level and why it is considered appropriate | Revising existing content | Added augmentations into item 26 |
| | b. 2.7.3 not rated as it depends on definition if vulnerable group; depends on the aims of the study e.g. when it is about access it surely should be reported on | Revising existing content | Added augmentations into items 18, 27, 31 |
| | c. Some of the aspects listed might be the questions of the evaluation and cannot be reported in the theoretical background but I realize that I am a little confused about what evaluation actually means, I understand it is not about measuring health outcomes but refining or developing the DHI." | Others | |
| | d. 2.4 & 2.6: These concepts are important, but should be reported as part of the methodology, not the theoretical background. | Restructuring existing content | Relocated to Methods section |
| | e. 2.7.1: It is important that the extent of participation is reported. However, I would not refer to specific models as the field of participatory design is very broad. For example, it seems that the particular model referenced to is only used in German settings. Additionally, forcing a theoretical model where a simple description of the extent of involvement would suffice, could limited the participation of people without formal research training in the writing/analytical process. | Revising existing content | Rewording of item 18 |
| | f. 2.73.: It is important to report on the extent that people in vulnerable situations were involved in the research. However, not every PD project involves people in vulnerable situations. Additionally, there may be other ethical concerns next to lack of representation of people in vulnerable situations. I'd suggest to formulate the criterium in a broader sense and tie this in with a stakeholder analysis. For example: ""outlining which people, including people in vulnerable situations, are stakeholders in the digital health intervention, and how the equitable participation of all relevant stakeholders was considered."" | Revising existing content | Rewording of item 18 |
| | g. 2.8.3 This item seems to touch on multiple topics. I'd separate this into several categories; target group, implementation plan, adoption plan. Also, keep in mind that very early formative evalutation may not | Revising existing content | Rewording of items 7, 18, 19 and 20 |

| | | | | |
|---|---|---|---|---|
| | | have a implementation plan in place yet, as this still need to be cocreated with the stakeholders. Therefore, any item pertaining to implementation and adoption should be considered optional. | | |
| | h. | 2.8.4 The examples are very closely related to the UX design of specifically web-based/app-based digital health technologies. Other health technologies (e.g. VR, sensing technology) may not be accurately represented with these examples. In addition, I'd again suggest here for the author not to ensure that the DHI does not vary in quality, but rather demonstrate to what extent the DHI takes diversity in stakeholders into account and make explicit any equity concerns that may have arised in their attempt to take the user into account. | Revising existing content | Rewording of item 18 |
| | i. | 2.8.5 Good idea, but the mention of a trial and version number assumes a static evaluation, which is opposite to the philosophy of most participatory research processes. For this item, I'd rather assume iteration than give the impression to authors that iteration in a participatory process is somehow faulty or a challenge to replicability (which is not an important quality criterium for this type of dynamic research anyhow). | | Added augmentations into item 20 |
| | j. | 2.8.6. I don't understand what you mean by call to action in this context." | Others | . |
| | k. | na | Others | . |
| | l. | no | Others | . |
| | m. | 2.6 and maybe 2.5 should be covered in the methods section | Restructuring existing content | Relocated to Methods section |
| | n. | "Before I go into more detail: Even though the envisioned chapter is to be called ""theoretical background"", no item refers to theories or frameworks (see above). Even if such an item should be added, there would still be lots more information in this section if any of the items made the cut, so I'd urge you to simply call the chapter ""background"". | Revising existing content | Added augmentation to topic of items 7 & 10 |
| | o. | In 2.2, I would remove statements on the intended impact, as this is an issue for the discussion rather than the background, imho. Therefore, I voted 4. | Revising existing content | Reworded item 7 |
| | p. | 2.4 belongs into the methods section, imho. As I don't know whether it will reappear there, I rated it important here. Let me please stress that this information is essential even when no survey design was used, as your item suggests otherwise. Qualitative research requires ethical approval as well." | Restructuring existing content | Relocated to Methods section |
| | q. | Re vulnerable populations, aspects of the above may not be completely appropriate depending on the context of the DHI | Revising existing content | Rewording of item 18 |
| | r. | I would see most of these points rather in the method section than in the theoretical background. As such, I found it tricky to weigh the importance of the content mentioned in sections 2.7-2.8 as I deem all of these as important - however, I would not expect them in the theoretical background | Restructuring existing content | Relocated and merged to items into Methods section |
| | s. | I do feel it is missleadin to if in 2.7.1 only on Framework is mentioned at it is one that is not used in the Health System Research but more in community health. Better naming some more or leave it out. | | Added augmentation to item 15 |
| | t. | No | Others | |
| | u. | 2.6 PARTICIPATION CONCEPT could be mentioned in methods. | Restructuring existing content | Content is covered by items 25 |
| | v. | 2.4 Ethical approval and 2.6 Participation concept are relevant for the method section, but not the theoretical background. | Restructuring existing content | Content is covered by items 25 |
| | w. | Items reported in the methods should be relevant to the study objectives. Some items could be left out as and when appropriate. | Revising existing content | . |
| | x. | no | Others | |
| | y. | "check Benton & Johnson, 2015 | Others | |
| | z. | include sensory difficulties (hypo or hyper sensoriality) | Revising scope | . |
| | aa. | I don't find Wright et al. | Others | |

| | | | | |
|---|---|---|---|---|
| | bb. | It should be important to check https://www.equator-network.org/" | Others | |
| | cc. | n/a | Others | |
| | dd. | I think all the elements that you mentioned are very important; I guess we need a balance of including all elements and the word limit of the report. Some information can be put in the appendix. | Revising scope | Added augmentation into item 10. Noted as general orientation for proper handling of the guideline. |
| | ee. | I believe some items could also be reported in the method section, e.g. study concept, participation concept. | Restructuring existing content | Relocated items 11, 13, 26, 31, |
| | ff. | There is often a separation between consumers as patients, and consumers as clinicians. There might be value in a specific denotation of this distinction as they have very different approaches and risks. | Revising existing content | Reworded throughout all items |
| | gg. | None | Others | |
| 5. Comments regarding missing items in Method section | a. | na | General approval | |
| | b. | It is very theoretical but very important. | General approval | |
| | c. | Reporting if AI was used to analyze data. | Revising scope | Added new item 74 |
| | d. | "Briefly describe any material used to support the evaluation (e.g. slides to explain concepts, templates to be filled out by participants) and also the followed dynamics (e.g. participants were grouped into teams, worked collaboratively or individually, predefined tasks to be completed, etc.) | Revising scope | Added new item 70 |
| | e. | The role of the researchers in the evaluation is also important: Were they just observed? How many researchers were involved in the evaluation session? Where the session was conducted (lab or real-settings)? Did researchers provide any support during the session?" | Revising existing content | Added new item 69 |
| | f. | 3.5.1 should be "describing and referencing the methods used" (methods literature is key in reporting of PD processes, imho) | Revising existing content | Reworded item 25 |
| | g. | describe if different languages have been used to include vulnerable groups. | Revising existing content | Content is covered by items 13 |
| | h. | Very often studies fail in terms of translation into the real world context. Very often the key issue is not the methodology itself, but describing well the context where it will be later on implemented. For example, DHI studies very often put in the consent "being able to read well English" but then in the reality that doesn't apply to all patients. Of DHI provide wearables, but that will not be feasible in the implementation. | Revising existing content | Reworded item 8 |
| | i. | No | General approval | |
| | j. | As mentioned in the abstract, it would be important to ensure reporting on representation of the study population in the design, technology development, policy-related aspects of the digital health intervention. These things have a significant impact on how well an intervention is adopted and used as well as associated outcomes. Things like gender analysis and/or taking a gender-intentional approach to research should be encouraged and documented. | Others | Noted as general orientation for proper handling of the guideline. |
| | k. | i understand that this is important but very theoretical | General criticism | |
| | l. | 3.5.2 describing the level or nature of participation used at various stages of the study. Refer to established stage models of participation (e.g., Wright et al.) - it is furthermore relevant which population was asked and in which setting. | Revising existing content | Reworded items 27, 38, 41, 43 |
| | m. | no | General approval | |
| | n. | reasons behind the chosen evaluation methods and the level of evidence of the method. Cite how it was validated | Revising existing content | Added augmentation in item 28 |
| | a. | "3.2. I doubt if CONSORT flow-charts can accurately represent very dynamic participatory research projects. Any visualization should suffice. | Revising existing content | Added augmentation in item 22 |

| | | | | | |
|---|---|---|---|---|---|
| 6. | Further comments regarding Method section | b. | 3.3. Assumes that the participatory evaluation was a survey, which is likely not the case. | Revising existing content | Reworded throughout all items by replacing survey with study |
| | | c. | 3.5.2 Similar comment as my earlier comment regarding the use of established models. | Revising scope | Merging of items 13 and 26 |
| | | d. | 3.5.6. I don't know what is meant with exclusion in this context. Exclusion of participants? model? methods? | Revising existing content | Rewording item 30 |
| | | e. | 3.5.7 Similar comment as my earlier comment regarding the inclusion of people in vulnerable situations. | Revising scope | Merging items 14 and 31 |
| | | f. | 3.6.1 & 2 These items are vaguely formulated. | Revising existing content | . |
| | | g. | 3.6.3 I do agree that it should be mentioned if instruments were specifically designed for the study, but assuming that they underwent the extent of the testing described in the item is not reasonable for a participatory design study. | Revising existing content | Rewording item 34 |
| | | h. | 3.7.3 ""the right people"" is a very vague description. | Revising existing content | Rewording item 38 |
| | | i. | 3.8.3 Assumes a survey as evaluation method. | Revising existing content | Reworded throughout all items by replacing survey with study |
| | | j. | 3.8.5. Not every drop-out requires counteracting. Especially people in vulnerable situations may have very reasonable reasons to drop-out of a study. Assuming that this is something necessary to counteract can be an inequitable act. Describe more neutrally (describing the number of drop-outs, and how these were responded to)." | Revising existing content | Reworded item 43 |
| | | k. | na | Others | |
| | | l. | With all included items method sections of manuscripts will be very long. | Revising scope | Noted as general orientation for proper handling of the guideline. |
| | | m. | no | Others | |
| | | n. | why the focus on surveys? | Revising existing content | Reworded throughout all items by replacing survey with study |
| | | o. | No | Others | |
| | | p. | no | Others | |
| | | q. | I don't completely understand this item: 3.5.6 explaining the reasons for the exclusion briefly, if established methods are deemed inappropriate or inapplicable for the specific context of the DHI being evaluated. | Revising existing content | Reworded item 30 |
| | | r. | At some point I gave other questions less coz I Valued the previous questions more. You know the likatt scale Effect. | Others | |
| | | s. | when I score 4, I mean the item can be put in the appendix. | Revising scope | Added note that some informations can be put to Information |
| | | t. | I did not understand 3.6.2 | Revising existing content | . |
| 7. | Comments regarding missing items in Results section | a. | na | Others | |
| | | b. | "Here's a much shorter version of potential key items for your ""Results"" section: 1. **Stakeholder Feedback**: Summarize feedback from patients, providers, and community members on the DHIs. | Revising scope | No revision, because proposed intense shortening does not reached overall consensus within the authors team. If this suggestion |

| | | | |
|---|---|---|---|
| | | | were to be taken, relevant aspects would not be covered. |
| | c. "Here’s a much shorter version of potential key items for your ""Results"" section: 2. **Health Outcomes**: Present key improvements in patient health or access to care from using DHIs. | Revising scope | No revision, since there was insufficient consensus among the authors team on this issue. Should this suggestion be implemented, it would result in a lack of coverage of relevant aspects. |
| | d. "Here’s a much shorter version of potential key items for your ""Results"" section: 3. **Usability**: Briefly describe how user-friendly and accessible the DHIs were for different groups. | Revising scope | No revision, since there was insufficient consensus among the authors team on this issue. Should this suggestion be implemented, it would result in a lack of coverage of relevant aspects. |
| | e. "Here’s a much shorter version of potential key items for your ""Results"" section: 4. **Challenges**: Mention any barriers, like technological issues or adoption challenges. | Revising scope | No revision, since there was insufficient consensus among the authors team on this issue. Should this suggestion be implemented, it would result in a lack of coverage of relevant aspects. |
| | f. "Here’s a much shorter version of potential key items for your ""Results"" section: 5. **Scalability**: Note if the DHIs can be applied on a larger scale or sustained long-term. | Revising scope | No revision, since there was insufficient consensus among the authors team on this issue. Should this suggestion be implemented, it would result in a lack of coverage of relevant aspects. |
| | g. If possible, results should include a relevance or significance analysis of the findings. | Revising existing content | Added augmentation into item 45 |
| | h. I was missing an item concerning the reporting of results for all methodological steps. I'd like it if your tool could thereby address selective reporting, in accordance with other RoB tools. | Revising scope | Added new item 71 |
| | i. No | General approval | |
| | j. I like that. | General approval | |
| | k. Also report of single data like individual cases. | Revising existing content | . |
| | l. no | General approval | |
| 8. Further comments regarding Results section | a. I feel that most aspects so far are pretty common sense of reporting research in general, I would appreciate if a reporting guide would only outline aspects specific to participation. | Revising existing content | Added augmentation into item 45 |
| | b. General note, this section diverges a bit in language used to describe participants, stakeholders, humans, researchers, stakeholders. Ideally, the guideline should use one umbrella term for everyone involved in the participatory approach. I'd suggest to engage a little bit more with the recent discussion regarding the use of these words, eg. https://link.springer.com/article/10.1007/s11625-024-01496-4 or https://www.fasttrackimpact.com/post/why-we-shouldn-t-banish-the-word-stakeholder | Revising existing content | Reworded throughout all items |

| | | | |
|---|---|---|---|
| | c. na | Others | |
| | d. 4.4 could be part of the limitations / strength section. | Restructuring existing content | Reworded item 48 |
| | e. non | | |
| | f. "Variables" in 4.1 again suggests you only address surveys as a means for PD. | Revising existing content | Reworded throughout all items by replacing survey with study |
| | g. not clear what you mean by ""type of assistance offered"" in 4.2 | Revising existing content | Reworded item 46 |
| | h. 4.3 is, of course, highly relevant, yet belongs into the discussion section, imho" | Revising scope | Merging items 47 and 58 |
| | i. 4.1 Also unexpected and/or negative results should be reported. | Revising scope | Added augmentation to new item 71 |
| | j. No | Others | |
| | k. 4.3 Impact of participation is more important for the discussion section. | Restructuring existing content | Relocated content to item 58 in Discussion section |
| | l. no | Others | |
| 9. Comments regarding missing items in Discussion section | a. na | Others | |
| | b. **Stakeholder Impact**: Evaluate the role of patient and citizen engagement in shaping DHIs. | Revising scope | No revision, since there was insufficient consensus among the authors team on this issue. Should this suggestion be implemented, it would result in a lack of coverage of relevant aspects. |
| | c. **Limitations**: Mention any challenges or biases in the participatory approach. | Revising scope | No revision, since there was insufficient consensus among the authors team on this issue. Should this suggestion be implemented, it would result in a lack of coverage of relevant aspects. |
| | d. **Comparison**: Briefly compare your findings to existing studies on DHIs. | Revising scope | No revision, since there was insufficient consensus among the authors team on this issue. Should this suggestion be implemented, it would result in a lack of coverage of relevant aspects. |
| | e. **Future Implications**: Highlight the potential influence on digital health design and policy. | Revising scope | No revision, since there was insufficient consensus among the authors team on this issue. Should this suggestion be implemented, it would result in a lack of coverage of relevant aspects. |

| | | | | |
|---|---|---|---|---|
| | f. | **Scalability**: Reflect on whether the DHIs can be scaled or sustained long-term. | Revising scope | No revision, since there was insufficient consensus among the authors team on this issue. Should this suggestion be implemented, it would result in a lack of coverage of relevant aspects. |
| | g. | "Impact of participation" should be placed here rather than in the results section. | Restructuring existing content | Relocated content from Result to Discussion section. Merging items 47 and 58 |
| | h. | No | General approval | |
| | i. | I don't understand this - and i want to see teh results. | Others | |
| | j. | Changes after the study, changes of people who were involved in the study and also changes of people who lead the study. | Revising scope | Rewording item 57 |
| | k. | no | General approval | |
| | l. | Related to 5.8.1, it would | Others | |
| | m. | na | Others | |
| 10. Further comments regarding Discussion section | a. | The order of items is uncommon, I'd suggest to move the limitations & generalizability after implications and results in relation to other studies. | Restructuring existing content | Resorting of items within section |
| | b. | 5.8: Again, this section makes some assumptions regarding the study which may go against the nature of an iterative participatory process, as outlined in my earlier comments." | Revising scope | Reworded items 57 & 60 |
| | c. | na | Others | |
| | d. | non not really | Others | |
| | e. | 5.8.5 I think barriers and facilitators for the reach of participants and adoption by organizations are mentioned the first time here. If they should be included in the discussion, it should be stated clearly, if they were examined systematically by applying respective methods, or if only assumptions of the researchers are discussed. Ideally, the study design involves the systematic evaluation of barriers and facilitators | Revising existing content | Added augmentation to item 24 |
| | f. | anything that has to do with "reporting" is very important, items involving "commenting" tend to be more subjective | Revising existing content | Reworded throughout all items |
| | g. | No | Others | |
| | h. | Some of the items might not be relevant, depending on the nature of the study or the digital health intervention | Revising scope | It is in the nature of the Delphi method that this is resolved |
| | i. | no | Others | |
| | j. | 1) "5.6 LESSONS LEARNED: Describe any lessons learned from the paticipatory evaluation of DHI that could be used to improve future evaluations and outcomes. Reflect on the things that went well and those that did not (e.g. success factors, challenges or budget considerations)" is not necessary as likely covered in 5.1-5.5. 2) "5.8.5 mentioning barriers or facilitators to the adoption of the intervention among study participants. Relates to individual-level structural, economic and social barriers or facilitators to access such as affordability, and other factors that may limit a user's ability to adopt the intervention" is crucial! | Revising scope | Item 55 is kept because it reached consensus |
| | k. | I might have been inclined to some aspects based on my professional background so I could be biased to value certain things more than others. | Others | |
| | l. | Section 5.1-5.7 feels just liken generic best practice academic writing. Essential but should not be specific to DHI reports. | Revising existing content | Rewording items 51, 53, 57 and 60 |

| | | | |
|---|---|---|---|
| 11. Comments regarding missing items in Others section | a. 6.3 report if participating citizens or patients did co-author | Revising existing content | Adding augmentation to item 64 |
| | b. Next to contributions of the authors, it would also be good to provide a description of the contribution of team members that are not authors. Sometimes citizens do not seek authorship while they have made important contributions. | Revising existing content | Adding augmentation to item 65 |
| | c. na | Others | |
| | d. no | General approval | |
| | e. Acknowledgements to people or entities who support or help in the study | Revising existing content | Adding augmentation to item 65 |
| | f. Ethics approval, if not reported in background or methods section | Restructuring existing content | . |
| | g. If the DHI is going to be commercialised by a company (startup, spin-off, etc) | Revising scope | Adding new item 72 |
| | h. Some types of publications (e.g., short papers) might not be long enough to cover all the aspects asked here. | Revising scope | Noted as general orientation for proper handling of the guideline. |
| | i. No | General approval | |
| | j. very important for me - because i want to know why you are doing the study and who benefits from it | General approval | |
| 12. Further comments regarding Others section | a. na | Others | |
| | b. No | Others | |
| | c. no | Others | |
| | d. 6.3 CONTRIBUTIONS: Share a detailed description of the contributions of the authors to the published work (e.g. using CRediT) | Revising existing content | Added augmentation to item 64 |
| 13. Comments for including reference examples | a. It helps to understand what is meant to be a reference. | Better understandig expectations. Clarity. | |
| | b. it facilitates your own scientific work. | Better understandig expectations. Clarity. | |
| | c. It might inspire other researchers to use those references. Yet, the reporting list needs to be updated continuously when references are updated. | Encourages use of frameworks/references/etc. | |
| | d. Ease of use it and clarity. | Better understandig expectations. Clarity. | |
| | e. they provide guidance on how to answer the question(s), provided, however, they are evidence-based. | Encourages use of frameworks/references/etc. | |
| | f. it is helpful for researchers, and it also improves comparability between studies and facilitates systematic reviews. | Encourages use of frameworks/references/etc. | |
| | g. you do not need to reinvent the wheel in this field. | Encourages use of frameworks/references/etc. | |
| | h. referencing established frameworks makes it easier to understand the procedure and to demonstrate the scientific correctness of the work. | Better understandig expectations. Clarity. Encourages use of frameworks/references/etc. | |
| | i. you can benefit from knowing also the others. | Knowledge expanding of existing Frameworks. | |
| | j. To make it clearer what is expected. | Better understandig expectations. Clarity. | |
| | k. Sometimes the question or the granularity only becomes clear this way. | Better understandig expectations. Clarity. | |
| | l. Makes expectations more explicit. | Better understandig expectations. Clarity. | |
| | m. could support a clear standard and socially robust quality. | Knowledge expanding of existing Frameworks. Better understandig expectations. Clarity. | |
| | n. it is easier to fill in with examples. | Better understandig expectations. Clarity. | |
| | o. It's always helpful to provide guidance so that the researchers know what they should be using to design and report on study findings. This will lead to better quality research and publications. | Better understandig expectations. Clarity. Knowledge expanding of existing Frameworks. | |
| | p. of transparency. | Better understandig expectations. Clarity. | |
| | q. It helps to clarify why they are important to report | Better understandig expectations. Clarity. | |

| | | | |
|---|---|---|---|
| | r. | references could be given more clearly. | Better understandig expectations. Clarity. |
| | s. | Enables the Reader to follow through. | Better understandig expectations. Clarity. |
| | t. | ...it makes it more tangible. | Better understandig expectations. Clarity. |
| | u. | the acronym could indicate different terms. | Better understandig expectations. Clarity. |
| | v. | Providing example will enhance standard formality. | Better understandig expectations. Clarity. |
| | w. | not everyone knows that especially people who are not expert in ID field | Encourages use of frameworks/references/etc. |
| | x. | Yes, examples of reference options (e.g., WHO) should be given. Including specific examples helps to clarify the intended standard or approach, making it easier for readers to understand, contextualize, and replicate the methodology. It also lends credibility to the work by showing alignment with established and reputable frameworks. | Better understandig expectations. Clarity. |
| 14. Comments against including reference examples | a. | As previously mentioned, existing frameworks may only be applicable to certain participatory studies (broad field) and be used by certain participants (those with formal research training). A description should suffice in those cases. | Limited applicability to specific context |
| | b. | There are a multitude of frameworks available which suit different projects. It may bias the selection of frameworks towards those listed on the reporting tool. It may be best left to peer reviewers to determine the suitability and use of any framework adopted within a study. | Limited applicability to specific context |
| | c. | this leads to answers which are very close to the example provided. | Others |
| | d. | This implies the supremacy of particular guidelines / frameworks and does not allow new frameworks to emerge or be promoted among the academic community. These frameworks should be constantly evolving, and therefore a higher level guideline such as this should not "pick winners". | Limited, wide-angle perspective on the spectrum of existing guidelines and frameworks. |
| | e. | I think this is an emerging field and the tension between rigor and innovation is difficult. True qualitative methods may not fit will with a reference option that has been previously validated. | Limited applicability to specific context |

## Supplementary Information S5: Detailed Results 2. Round

| OID | Item Titel | Item- full text | 1- Very unim-portant | 1 (%) | 2 | 2 (%) | 3 | 3 (%) | 4 | 4 (%) | 5- Very im-portant | 5 (%) | Can-not rate this item | Can-not rate this item (%) | CR (%) | Mean | SD | Consequences from qualitative comments |
|---|---|---|---|---|---|---|---|---|---|---|---|---|---|---|---|---|---|---|
| Abstract | | | | | | | | | | | | | | | | | | |
| 65 | Background | Briefly state the background and context of the study, including the state of the art. | 1 | 2.9 | 2 | 5.7 | 5 | 14.3 | 15 | 42.9 | 12 | 34.3 | 0 | 0.0 | 77.1 | 4.00 | 1.000 | Revised based on comment 1b, Supplementary Information S6 |
| 5 | Keywords | List relevant keywords that represent the core topics of the study | 0 | 0.0 | 15 | 22.7 | 12 | 18.2 | 19 | 28.8 | 22 | 33.3 | 0 | 0.0 | 62.1 | 3.67 | 1.141 | |
| Background & Foundation | | | | | | | | | | | | | | | | | | |
| 66 | DHI Setting | Describe the setting of the DHI and the real-world context where it will be later on implemented. Add information on the health domain it addresses, the specific medical issue it targets and the intended use setting. | 2 | 5.7 | 1 | 2.9 | 0 | 0.0 | 15 | 42.9 | 17 | 48.6 | 0 | 0.0 | 91.4 | 4.26 | 1.039 | |
| 67 | DHI Development | DHI Development | 2 | 5.7 | 0 | 0.0 | 7 | 20.0 | 13 | 37.1 | 13 | 37.1 | 0 | 0.0 | 74.3 | 4.00 | 1.057 | |
| | DHI Description | Describe the type of the DHI that is the object of the study by ... (If there is one or more comparators all the following points also have to be addressed) | | | | | | | | | | | | | | | | |
| 15 | DHI Description | a) referring to established frameworks or classification systems (e.g. those provided by the WHO/NICE) to classify DHI | 2 | 5.7 | 2 | 5.7 | 15 | 42.9 | 8 | 22.9 | 7 | 20.0 | 1 | 2.9 | 42.9 | 3.37 | 1.215 | |
| 19 | DHI Description Updating and revisions | e) mentioning the iterations of the DHI or describing whether the intervention underwent major changes during the evaluation process or | 2 | 5.7 | 2 | 5.7 | 9 | 25.7 | 16 | 45.7 | 6 | 17.1 | 0 | 0.0 | 62.9 | 3.63 | 1.031 | Revised based on comment 2d, Supplementary Information S6 |

| | | | | | | | | | | | | | | | | | |
|---|---|---|---|---|---|---|---|---|---|---|---|---|---|---|---|---|---|
| | | whether the development and/or content was "frozen" during the trial. | | | | | | | | | | | | | | | |
| 20 | DHI Description Tailoring during use | f) describing dynamic components (e.g. news feeds, changing content, usage of ongoing feedback based on the end-user's state and activities, personalization) which may have an impact on the replicability of the intervention. Describe whether there are any strategies to support tailoring the DHI to participants over time. | 2 | 5.7 | 2 | 5.7 | 7 | 20.0 | 13 | 37.1 | 10 | 28.6 | 1 | 2.9 | 65.7 | 3.69 | 1.278 | |
| Method | | | | | | | | | | | | | | | | | |
| 22 | Study flow | Visualize the research process (e.g. CONSORT flow chart or others). | 1 | 2.9 | 2 | 5.7 | 8 | 22.9 | 11 | 31.4 | 13 | 37.1 | 0 | 0.0 | 68.6 | 3.94 | 1.056 | Revised based on comment 3c, Supplementary Information S6 |
| 23 | Informed consent | Describe the informed consent process by where the participants were told the length of time of the study, which data were stored and where and for how long, who the investigator was, and what the purpose of the study is. Add information about the possibilities of drop-out | 1 | 2.9 | 4 | 11.4 | 8 | 22.9 | 9 | 25.7 | 13 | 37.1 | 0 | 0.0 | 62.9 | 3.83 | 1.150 | Relocation |
| 11 | Participation Concept | Describe what methodological orientation was stated to underpin the study, e.g. grounded theory, discourse analysis, ethnography, phenomenology, content analysis. | 1 | 2.9 | 0 | 0.0 | 1 | 2.9 | 13 | 37.1 | 20 | 57.1 | 0 | 0.0 | 94.3 | 4.46 | 0.817 | |
| 24 | Impacts/Effects of participation | If applicable, report the methods used to explore the impact of participation as well as barriers and facilitators of participation in the study. | 1 | 2.9 | 1 | 2.9 | 5 | 14.3 | 16 | 45.7 | 12 | 34.3 | 0 | 0.0 | 80.0 | 4.06 | 9.938 | |
| 74 | Usage of AI | Report if AI was used to analyze data. | 3 | 8.6 | 4 | 11.4 | 6 | 17.1 | 9 | 25.7 | 12 | 34.3 | 1 | 2.9 | 60.0 | 3.57 | 1.441 | |

| | | | | | | | | | | | | | | | | | | |
|---|---|---|---|---|---|---|---|---|---|---|---|---|---|---|---|---|---|---|
| | Participatory Evaluation Method | Explain the level or nature of participation and participatory evaluation methods used at various stages of the study by … | | | | | | | | | | | | | | | | |
| 29 | Participatory Evaluation Method | a) defining the used evaluation criteria including their underlying indicators by referring to existing frameworks (e.g. WHO, NICE, EXPH, the Swiss Evaluation framework by Kowatsch et al, the approach of Murray et al. or others). | 1 | 2.9 | 1 | 2.9 | 6 | 17.1 | 13 | 37.1 | 7 | 20.0 | 1 | 2.9 | 57.1 | 3.51 | 1.380 | |
| 30 | Participatory Evaluation Method | e) if established methods, frameworks or approaches are deemed inappropriate or inapplicable for the specific context of the DHI being evaluated, explaining the reasons for the exclusion briefly. | 2 | 5.7 | 2 | 5.7 | 11 | 31.4 | 16 | 45.7 | 4 | 11.4 | 0 | 0.0 | 57.1 | 3.51 | 0.981 | |
| | Data Collection & Measurements | Provide details on the data collection methods used for each variable of interest, by … | | | | | | | | | | | | | | | | |
| 33 | Data Collection & Measurements | b) explaining which roles established measurements play in the study. If established measurements can't be used, explain why. | 0 | 0.0 | 3 | 8.6 | 10 | 28.6 | 13 | 37.1 | 9 | 25.7 | 0 | 0.0 | 62.9 | 3.80 | 0.933 | |
| 35 | Data Collection & Measurements | d) describing the comparability for each chosen measurement. | 1 | 2.9 | 10 | 28.6 | 10 | 28.6 | 12 | 34.3 | 1 | 2.9 | 1 | 2.9 | 37.1 | 2.97 | 0.961 | |
| 68 | Data Collection & Measurements | e) describing the setting of the data collection as well as the dynamics (e.g. labor or real setting, participants were grouped into teams, worked collaboratively or individually, predefined tasks to be completed). | 1 | 2.9 | 1 | 2.9 | 4 | 11.4 | 11 | 31.4 | 18 | 51.4 | 0 | 0.0 | 82.9 | 4.26 | 0.980 | |
| 69 | Data Collection & Measurements | f) describing the role of the researchers in the evaluation (e.g. supporting role, silent observer). | 2 | 5.7 | 2 | 5.7 | 4 | 11.4 | 10 | 28.6 | 17 | 48.6 | 0 | 0.0 | 77.1 | 4.09 | 1.173 | |

| | | | | | | | | | | | | | | | | | | |
|---|---|---|---|---|---|---|---|---|---|---|---|---|---|---|---|---|---|---|
| | Sampling | Describe who participated by… | | | | | | | | | | | | | | | | |
| 38 | Sampling | c) explaining how it is ensured that the people who were involved were the people who fit the research question (e.g. stakeholder analysis, different digital health literacy levels). | 0 | 0.0 | 3 | 8.6 | 2 | 5.7 | 12 | 34.3 | 18 | 51.4 | 0 | 0.0 | 85.7 | 4.29 | 0.926 | |
| | Recruitment & Engagement Strategies | Describe in detail how participants were recruited, and which strategies were used to engage them by | | | | | | | | | | | | | | | | |
| 41 | Recruitment & Engagement Strategies | c) describing how, how often, and where the study was announced or advertised | 1 | 2.9 | 9 | 25.7 | 14 | 40.0 | 8 | 22.9 | 3 | 8.6 | 0 | 0.0 | 31.4 | 3.09 | 0.981 | |
| 42 | Recruitment & Engagement Strategies | d) reporting relevant dates, periods of recruitment and follow-ups | 3 | 8.6 | 5 | 14.3 | 8 | 22.9 | 14 | 40.0 | 5 | 14.3 | 0 | 0.0 | 54.3 | 3.37 | 1.165 | |
| 43 | Recruitment & Engagement Strategies | e) describing the number of drop-outs and how these were responded to. | 2 | 5.7 | 2 | 5.7 | 10 | 28.6 | 10 | 28.6 | 11 | 31.4 | 0 | 0.0 | 60.0 | 3.74 | 1.146 | |
| 70 | Recruitment & Engagement Strategies | h) describing any material used to support the engagement in the evaluation (e.g. slides to explain concepts, templates to be filled out by participants). | 1 | 2.9 | 5 | 14.3 | 8 | 22.9 | 18 | 51.4 | 3 | 8.6 | 0 | 0.0 | 60.0 | 3.49 | 0.951 | |
| 74 | Usage of AI | | 3 | 8.6 | 4 | 11.4 | 6 | 17.1 | 9 | 25.7 | 12 | 34.3 | 1 | 2.9 | 60.0 | 3.57 | 1.441 | |
| Results | | | | | | | | | | | | | | | | | | |
| 48 | Impacts/Effects of participation | If applicable, report shorty the measured impact as well as barriers and facilitators of participation | 1 | 2.9 | 1 | 2.9 | 9 | 25.7 | 11 | 31.4 | 12 | 34.3 | 1 | 2.9 | 65.7 | 3.83 | 1.200 | Revised based on comment 4a, Supplementary Information S6 |
| 71 | Selective Reporting | To avoid selective reporting and risk of bias, report if any findings (e.g. negative findings) or unexpected results are not detailed described. Explain why. | 1 | 2.9 | 5 | 14.3 | 5 | 14.3 | 12 | 34.3 | 11 | 31.4 | 1 | 2.9 | 65.7 | 3.69 | 1.301 | |

| | | | | | | | | | | | | | | | | | | |
|---|---|---|---|---|---|---|---|---|---|---|---|---|---|---|---|---|---|---|
| Discussion | | | | | | | | | | | | | | | | | | |
| | Reflecting participation | Discuss the process and the results of the participation by … | | | | | | | | | | | | | | | | |
| 57 | Reflecting participation | a) commenting, if it was possible to carry out the participation as planned. Reflect on what are the facilitators and barriers for participants to participate in the research and design process. | 1 | 2.9 | 0 | 0.0 | 5 | 14.3 | 16 | 45.7 | 13 | 37.1 | 0 | 0.0 | 82.9 | 4.14 | 0.879 | |
| 60 | Reflecting participation | d) If applicable, describing on the rigor of the method used to capture or measure the impact as well as barriers and facilitators of participation. | 1 | 2.9 | 0 | 0.0 | 14 | 40.0 | 12 | 34.3 | 8 | 22.9 | 0 | 0.0 | 57.1 | 3.74 | 0.919 | |
| 72 | Commercialization | Describe if the DHI is going to be commercialized by a company (e.g., startup, spin-off). | 4 | 11.4 | 4 | 11.4 | 7 | 20.0 | 7 | 20.0 | 12 | 34.3 | 0 | 0.0 | 54.3 | 3.46 | 1.502 | |
| Certainty | | | | | | | | | | | | | | | | | | |
| | Certainty Abstract | | 1 | 2.9 | 1 | 2.9 | 2 | 5.7 | 12 | 34.3 | 19 | 54.3 | 0 | 0.0 | 88.6 | 4.34 | | |
| | Certainty Background | | 0 | 0.0 | 1 | 2.9 | 7 | 20.0 | 14 | 40.0 | 13 | 37.1 | 0 | 0.0 | 77.1 | 4.11 | | |
| | Certainty Method | | 1 | 2.9 | 2 | 5.7 | 1 | 2.9 | 16 | 45.7 | 15 | 42.9 | 0 | 0.0 | 88.6 | 4.20 | | |
| | Certainty Results | | 1 | 2.9 | 1 | 2.9 | 2 | 5.7 | 16 | 45.7 | 15 | 42.9 | 0 | 0.0 | 88.6 | 4.23 | | |
| | Certainty Discussion | | 0 | 0.0 | 2 | 5.7 | 5 | 14.3 | 14 | 40.0 | 14 | 40.0 | 0 | 0.0 | 80.0 | 4.14 | | |
| | Certainly Others | | 0 | 0.0 | 1 | 1.5 | 5 | 14.3 | 12 | 34.3 | 17 | 48.6 | 0 | 0.0 | 82.9 | 4.29 | | |

## Supplementary Information 6: Qualitative Results 2. Round

| Origin free-text field | Comment | Category | Effect of comment |
|---|---|---|---|
| 1. Further comments regarding Abstract section | a. state of the art may be too much if abstract limited to 250 words. | Revising scope | |
| | b. Regarding the BACKGROUND section, for me it is important to state the context and the research gap. | Revising existing content | Added augmentation into item 65 |
| | c. Yes, I strongly believe that the list of KEYWORDS and topic background gives the reader the highlights of a given research publication. | General approval | |
| | d. Abstracts are critical for evidence synthesis through systematic review. A well crafted abstract may mean inclusion/exclusion of evidence. | Revising scope | |
| 2. Further comments regarding Background section | a. From my point of view, ethical vote and DHI description belong to the methods section. | Restructuring existing content | |
| | b. Contextual factors make or break success of interventions and are critical to understand when it comes to replicability. | Others | |
| | c. If possible, it should be mentioned what influence the findings of the study have."" --> unclear what study is referred to | Revising existing content | |
| | d. mentioning the iteration of the DHI"" --> does this item refer to the DHI development process? Should be phrased more precisely" | Revising existing content | 19 |
| 3. Further comments regarding Method section | a. Informed consent is important, but as part of a declarations section and not necessarily in the methods section itself. | Restructuring existing content | |
| | b. Impact of participation is more or less part of the discussion, because it is very difficult to operationalize this issue." | Restructuring existing content | |
| | c. With regard to study flow: any visualization should be sufficient for meeting this reporting requirement. Some more iterative approaches to PPI may not fit the constricts of static CONSORT-like flowcharts. | Revising existing content | Rewording item 22 |
| | d. The idea of 'drop-out' goes against the philosophical nature of equal participation and is therefore very unfit for participatory work. A more neutral criterium in relation to changing team composition (which may include researchers, citizens, or any other person part of the team) could potentially be fitting." | Revising existing content | |
| | e. These are qualitative methods: grounded theory, discourse analysis, ethnography, phenomenology, content analysis. | Revising existing content | Controlled item 11 |
| | f. Unclear what comparability refers to in measurement selection? What should measurements be comparable to? Each other, a benchmark...? | Revising existing content | Taken for discussion into workshop |
| | g. I was missing references to the target group of the THI in the sampling process. Sampling from potential users should be taken into account as well as sampling according to the research questions, which I like a lot, btw!" | Revising scope | Content is covered by items 39-44 & 13 |
| | h. some point you mention should be state of the art of sound research. I therefore ranked them in 3 | Revising scope | Content is covered by item 54 |
| 4. Further comments regarding Results section | a. Does the use of the word 'shorty' mean briefly? | Revising existing content | Rewording item 48 |
| | b. I guess the criterion "Selective reporting" will not work in practice. | General criticism | |
| | c. There is wide variability in reporting of results due to variation in measurements. In a quality checklist, it’s important to allow for this. | Others | |
| | d. Explaining why findings are not reported seems like adhering to the logic of publication processes (word limits, pressure to report positive/expected results) rather than to rigorous methodology. I emphatically advocate for simply reporting all results which are not intended for another publication. If the latter should be the case, then this should be reported as well | Revising existing content | Taken for discussion into workshop |

| | | | | |
|---|---|---|---|---|
| 5. Further comments regarding Discussion section | a. | How about discussing the benefit of participation? If it should be evaluated, as stated above, reflecting on whether participation yielded a benefit seems paramount to me. | Revising scope | Content is covered by item 55 |
| 6. Further comments regarding Others section | a. | could improve by adding 'If applicable/where applicable....' | Revising existing content | . |
| | b. | For most of my answer my rating of importance Was somehow influenced by my professional and passionate interests. For example the area I advocate For I of course give very important even when I avoid it. | Others | |
| | c. | Commercialisation is likely unknown or changed at the time of publishing, so would not be possible to make a mandatory reporting standard. | Revising existing content | Taken for discussion into workshop |
| 7. General Further Comments | a. | Yes, I would be interested to read about the final research findings of this study. | General approval | |
| | b. | I love your work | General approval | |

Supplementary Information S7: Results 3. Round

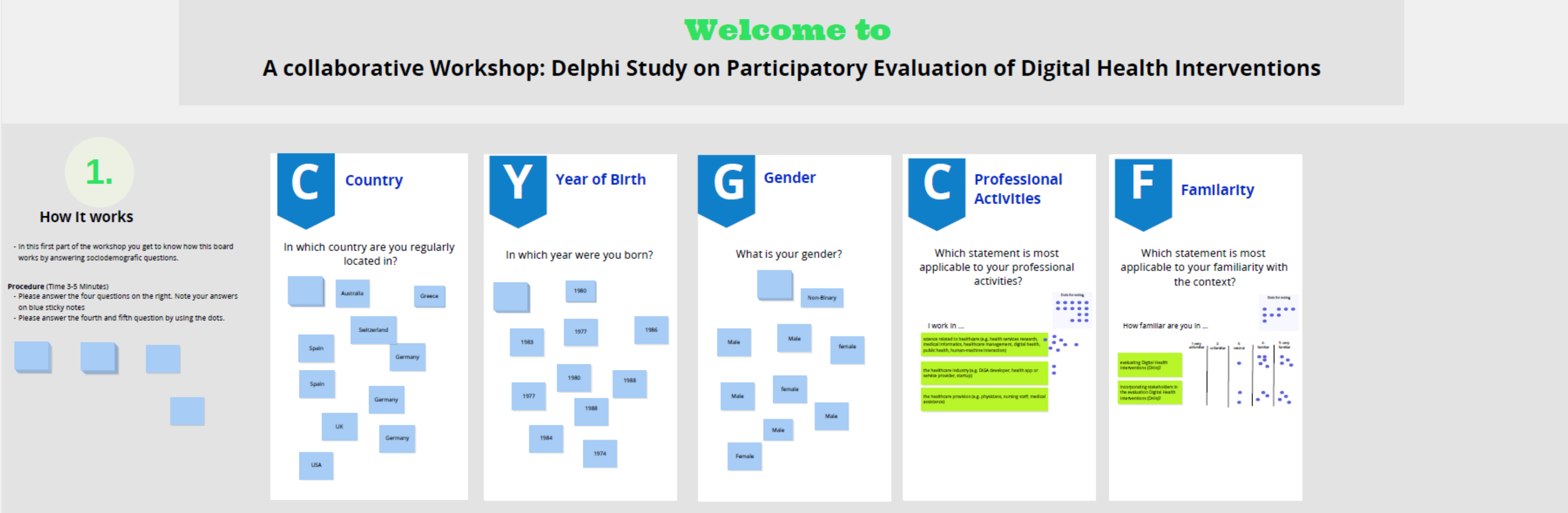

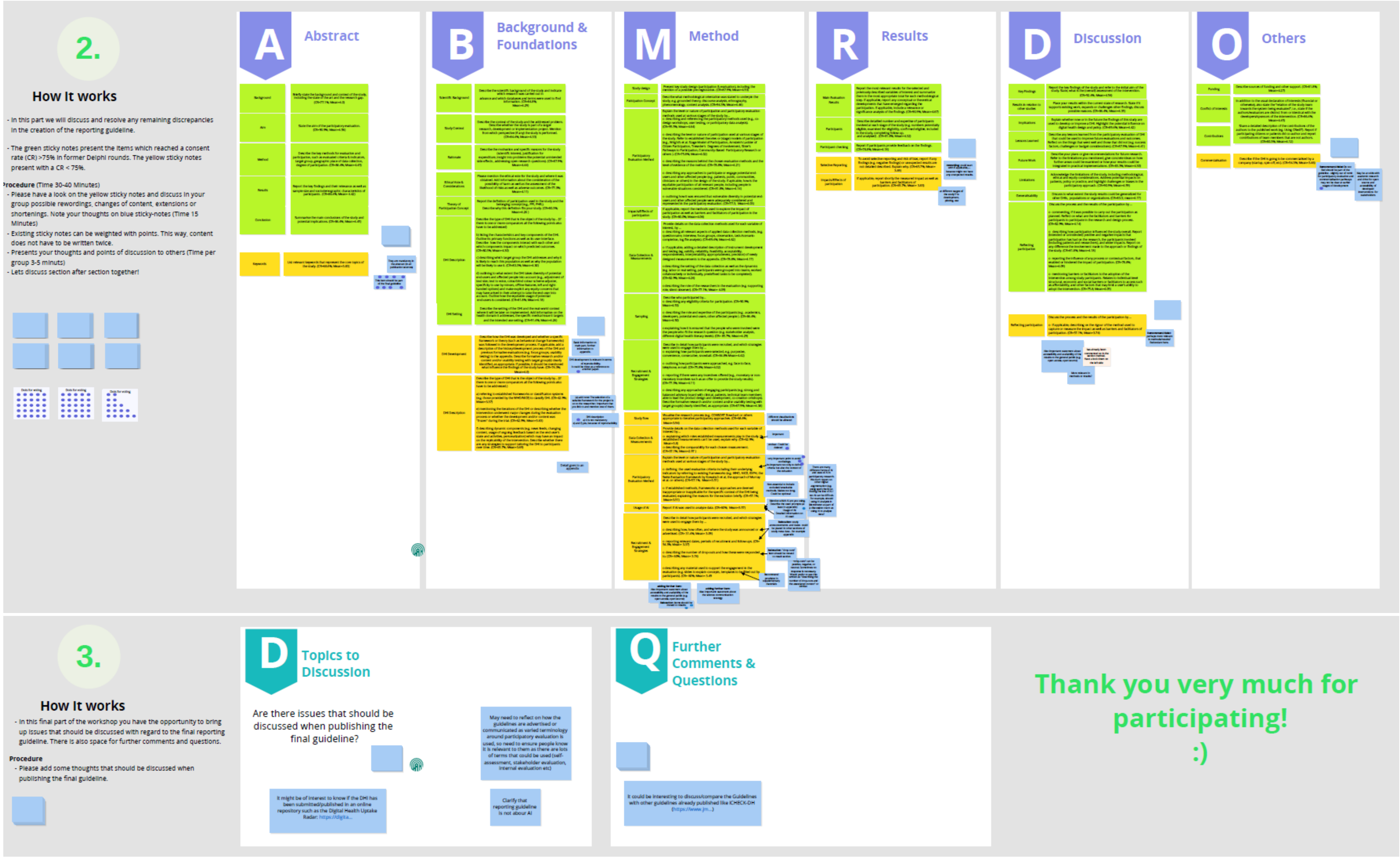
2.
How it works
- In this part we will discuss and resolve any remaining discrepancies in the creation of the reporting guideline.
- The green sticky notes present the items which reached a consent rate (CR) >75% in former Delphi rounds. The yellow sticky notes present with a CR < 75%.
Procedure (Time 30-40 Minutes)
- Please have a look on the yellow sticky notes and discuss in your group possible rewordings, changes of content, extensions or shortenings. Note your thoughts on blue sticky-notes (Time 15 Minutes)
- Existing sticky notes can be weighted with points. This way, content does not have to be written twice.
- Presents your thoughts and points of discussion to others (Time per group 3-5 minuts)
- Lets discuss section after section together!
A Abstract
B Background & Foundations
M Method
R Results
D Discussion
O Others
3.
How it works
- In this final part of the workshop you have the opportunity to bring up issues that should be discussed with regard to the final reporting guideline. There is also space for further comments and questions.
Procedure
- Please add some thoughts that should be discussed when publishing the final guideline.
D Topics to Discussion
Are there issues that should be discussed when publishing the final guideline?
May need to reflect on how the guidelines are advertised or communicated as varied terminology around participatory evaluation is used, so need to ensure people know it is relevant to them as there are lots of terms that could be used (self-assessment, stakeholder evaluation, internal evaluation etc)
It might be of interest to know if the DHI has been submitted/published in an online repository such as the Digital Health Uptake Radar: https://digita...
Clarify that reporting guideline is not abour AI
Q Further Comments & Questions
It could be interesting to discuss/compare the Guidelines with other guidelines already published like ICHECK-DH (https://www.jm...)
Thank you very much for participating!
:)

Supplementary Information S8: DELPHISTAR Checklist

## Delphi studies in social and health sciences – recommendations for an interdisciplinary standardized reporting (DELPHISTAR)

This reporting guideline is meant for studies using Delphi techniques in the health and social sciences. These also include all Delphi variants and modifications that meet the following criteria:

1. Survey of several people with specialized knowledge (e.g., operational knowledge, experiential knowledge, functional knowledge, contextual knowledge);
2. Structured communication process that involves a group of people with relevant expertise;
3. Carrying out at least two survey rounds or the option to respond at least two times;
4. Feedback: the (interim) results are presented to the respondents starting in the second round;
5. Basis is a quantitative questionnaire with the possibility to contribute or supplement arguments for the respective position;
6. All answers, quantitative and qualitative, are systematically analyzed (quantitative: e.g., descriptive statistics, qualitative: e.g., thematic analysis).

| Topic | Section | Item | Checklist Item | Location where item is reported |
|---|---|---|---|---|
| I Title and Abstract | | 1 | Identification as a Delphi procedure in the title | Titel. Page 1 |
| | | 2 | Identification as a Delphi procedure in the abstract | Abstract- Method |
| | | 3 | Structured abstract | Abstract |
| II Context | Formal | 4 | Information about the sources of funding | Abstract |
| | | 5 | Information about the team of authors and/or researchers (e.g., discipline, institution) | Method- Overview |
| | | 6 | Information about method consulting | n/a |
| | | 7 | Information about the project background | Method- Overview |
| | | 8 | Information about the study protocol | Method- Overview |
| | Content | 9 | Justification of the chosen method (Delphi procedure) to answer the research question | Method- Overview |
| | | 10 | Aim of the Delphi procedure (e.g., consensus, forecasting) | Aim & Method- Overview |
| III Method | Body & Integration of knowledge | 11 | Identification and elucidation of relevant expertise, spheres of experience, and perspectives (e.g., theory, practice, affected groups, disciplines) | Method- Selection of participants |
| | | 12 | Handling of knowledge, expertise and perspectives which are missing or have been deliberately not integrated | Method- Selection of participants |
| | | 13 | Basic definition of expert[1] | Method- Selection of participants |
| | Delphi variations | 14 | Identification of the type of Delphi procedure and potential modifications (e.g., classic Delphi, real-time Delphi, group Delphi) | Method- Delphi Procedure & Data Collection |
| | | 15 | Justification of the Delphi variation and modifications, including during the Delphi process, if applicable | n/a |
| | Sample of experts | 16 | Selection criteria for the experts (per round if there are different expert groups) | Method- Selection of participants |
| | | 17 | Identification of the experts | Method- Selection of participants |
| | | 18 | Information about recruiting and any subsequent recruiting of experts | Method- Selection of participants |
| | Survey | 19 | Elucidation of the content development for the questionnaire[2] | Method- Delphi Procedure & Data Collection |
| | | 20 | Description of the questionnaire (content and structure) | Method- Delphi Procedure & Data Collection |
| | Delphi rounds | 21 | Number of Delphi rounds | Method- Overview |
| | | 22 | Information about the aims of the individual Delphi rounds | Method- Delphi Procedure & Data Collection |
| | | 23 | Disclosure and justification of the criterion for discontinuation | Method- Delphi Procedure & Data Collection |
| | Feedback | 24 | Information about what data was reported back per round | Method- Delphi Procedure & Data Collection |
| | | 25 | Information on how the results of the previous Delphi round were fed back to the experts surveyed (e.g., via | Method- Delphi Procedure & Data Collection |

| Topic | Section | Item | Checklist Item | Location where item is reported |
|---|---|---|---|---|
| | | | frequencies, mean values, measures of dispersion, listing of comments) | |
| | | 26 | Information on whether feedback was differentiated by specific groups (e.g., by field of expertise, institutional affiliation) | Method- Delphi Procedure & Data Collection |
| | | 27 | Information about how dissent and unclear results were handled | Method- Delphi Procedure & Data Collection |
| | **Data analysis** | 28 | Disclosure of the quantitative and qualitative analytical strategy | Method- Analysis |
| | | 29 | Definition and measurement of consensus | Method- Analysis |
| | | 30 | Information on group-specific analysis or weighting of experts (e.g., theory vs. practice, discipline-specific analysis) | n/a |
| **IV Results** | **Delphi process** | 31 | Illustration of the Delphi process (e.g., in a flow chart) | Figures 1 and 3 |
| | | 32 | Information about special aspects during the Delphi process (e.g., deviations from the intended approach with justification) | n/a |
| | | 33 | Number of experts per round (both invited and participating) | Results- Panel Characteristics; Table 1 and Figure 2 |
| | **Results** | 34 | Presentation of the results for each Delphi round and the final results | Results; Supplementary Information 2 to 7 |
| **V Discussion** | **Quality of findings** | 35 | Highlighting the findings from the Delphi study | Discussion |
| | | 36 | Validity of the results (e.g., transferability of the findings) | Discussion |
| | | 37 | Reliability of the results (e.g., split half, inter-rater reliability) | Method- Analysis |
| | | 38 | Reflection on potential limitations (e.g., distortion, skewing, bias) | Discussion- Limitations |

[1] "Experts" are the participants; this can be people from academia, practice, or representatives of lived experience (e.g., patients, family members).
[2] The term "questionnaire" stands for the survey instrument regardless of whether quantitative or qualitative items are integrated or weighted.

### Contact

Prof. Dr. Marlen Niederberger
E-mail: marlen.niederberger(at)ph-gmuend.de
Department of Research Methods in Health Promotion and Prevention, Institute for Health Sciences
University of Education Schwäbisch Gmünd, Oberbettringer Straße 200, 73525 Schwäbisch Gmünd, Germany

Supplementary Information S9: Final Reporting Guideline

## Reporting Guideline for the Participatory Development and Evaluation of Digital Health Interventions (ParDE-DHI)

Explanation for usage

- This reporting guideline provides a set of recommendations on information that should be included for a comprehensive reporting of participatory development and evaluation of Digital Health Interventions (DHIs). This also includes guidance on how some information might best be presented and lists of possible frameworks for reference. The selection of appropriate formats and frameworks is up to the researcher and can be something that is not listed as an example.
- ParDE-DHI focuses on the development and evaluation of DHIs. Despite the comprehensive and holistic nature of the development process, it may be necessary to consider additional reporting guidelines in conjunction with it (e.g. TIDieR checklist for intervention description). This decision is up to the researchers.
- Each journal has different requirements, so some items may be required in other sections. This should not be irritating. Authors can change the order of the items if they want to keep the items under the subheadings.
- Not all items can be fully addressed in the main body of a publication, so some items include a note to add information to the appendix. Alternatively, reference may be made to other existing or planned publications that address the issue.

| No. | Section and Topic | Item/Guidance for Reporting | Location where item is reported/ Reported on page |
|---|---|---|---|
| | **ABSTRACT** | | |
| 1 | Background | Briefly state the background and context of the study, including the state of the art and the research gap. | |
| 2 | Aim | State the aim of the participatory development and evaluation. | |
| 3 | Method | Describe the key methods for evaluation and participation, such as evaluated criteria & indicators, target group, geographic place of data collection, and degree of participation. | |
| 4 | Results | Report the key findings and their relevance, as well as the sample size and sociodemographic characteristics of participants. | |
| 5 | Conclusion | Summarize the main conclusions of the study and potential implications. | |
| 6 | Keyword | List relevant keywords that represent the core topics of the study. | |
| | **BACKGROUND & FOUNDATION** | | |
| 7 | Scientific Background | Describe the scientific background of the study and indicate which research was carried out in advance and which databases and terms were used to find information. | |
| 8 | Study Context | Describe the context of the study and the addressed problem. Describe whether the study is part of a larger research, development, or implementation project. Mention from which perspective (if any) the study is performed. | |
| 9 | Rationale | Describe the motivation and specific reasons for the study (scientific interest, justification for expenditure, insight into problems like potential unintended side effects, addressing open research questions). | |
| 10 | Ethical Vote & Considerations | Please mention the ethical vote for the study and where it was obtained. Add information about the consideration of the possibility of harm, as well as the assessment of the likelihood of risks, as well as adverse outcomes. | |
| 11 | Theory of Participation Concept* | Report the definition of participation used in the study and the belonging concept (e.g., PPI, PHR). Describe why this definition fits your study. | |

| | | | |
|---|---|---|---|
| 12 | Digital Health Intervention (DHI) Setting* | Describe the setting of the DHI and the real-world context where it will be implemented later on. Add information on the health domain it addresses, the specific healthcare issue it targets, and the intended use setting. | |
| 13 | DHI Development* | Describe how the DHI was developed and whether a specific framework or theory (such as behavioral change frameworks or GUIDED recommendations) was followed in the development process. If applicable, add a description of the history/development process of the DHI and previous formative evaluations (e.g., focus groups, usability testing) to the appendix. Describe formative research and/or content and/or usability testing with target group(s) clearly identified, as appropriate. If possible, it should be mentioned what influence the findings of the study have. | |
| 14 | DHI Description | Describe the type of DHI that is the object of the study by ...<br>(If there is/are one or more comparators, all the following points also must be addressed.)<br>a) referring to established frameworks or classification systems (e.g., TIDieR-Checklist or those provided by the WHO or NICE) to classify the DHI. The selection of an appropriate framework for the project is up to the researcher.<br>b) listing the characteristics and key components of the DHI. Outline its primary functions as well as its user interface. Describe how the components interact with each other, and which components impact which predicted outcomes.<br>c) describing which target group the DHI addresses and why it is likely to reach this population, as well as why the population will be likely to use it.<br>d) outlining to what extent the DHI takes diversity of potential end-users and affected people into account (e.g., adjustment of text size, text to voice, colorblind color scheme adjuster, specificity to use by minors, offline features, left and right-handed options) and make explicit any equity concerns that may have arised in their attempt to take the end-user into account. Outline how the equitable usage of potential end-users is considered.<br>e) mentioning the iterations of the DHI or describing whether the intervention underwent major changes during the evaluation process, or whether the development and/or content was "frozen" during the trial.<br>f) describing dynamic components (e.g., news feeds, changing content, usage of ongoing feedback based on the end-user's state and activities, personalization) which may have an impact on the replicability of the intervention. Describe whether there are any strategies to support tailoring the DHI to participants over time. | |
| | **METHOD** | | |
| 15 | Study Design | Present key study design (participatory development & evaluation), including the mention of a possible pre-registration. | |
| 16 | Study Flow | Visualize the research process (e.g., CONSORT flowchart or others appropriate to iterative participatory approaches). | |
| 17 | Informed Consent | Describe the informed consent process by which the participants were told the length of time of the study, which data were stored and where, and for how long, who the investigator was, the possibilities of drop-out, and what the purpose of the study was. | |
| 18 | Methodological Orientation | Describe what methodological orientation was stated to underpin the study, e.g., grounded theory, discourse analysis, ethnography, phenomenology, content analysis. | |
| 19 | Participatory Evaluation Method | Explain the level or nature of participation and participatory development and evaluation methods used at various stages of the study by ...<br>a) defining the used evaluation criteria, including their underlying indicators by referring to existing frameworks (e.g., WHO, NICE, EXPH, the Swiss Evaluation framework by Kowatsch et al., the approach of Murray et al., or others).<br>b) describing and referencing the participatory methods used (e.g., co-design workshops, user testing, or participatory data analysis).<br>c) describing the level or nature of participation used at various stages of the study. Refer to established theories or (stage) models of participation (e.g., Wright et al.'s Stage Model of Participation, Arnstein's Ladder of Citizen Participation, Treseder's Degrees of Involvement, Shier's Pathways to Participation, Community-Based Participatory Research, or others).<br>d) describing the reasons behind the chosen development and evaluation methods and the level of evidence of the method.<br>e) (optional) describing if established methods, frameworks, or approaches are deemed inappropriate or inapplicable for the specific context of the DHI being evaluated, explaining the reasons for the exclusion briefly.<br>f) describing any approaches to participate or engage potential end-users and other affected people (e.g., patients, public, communities, clinicians, or others) in the design of the study. If applicable, describe how the equitable participation of all relevant people, including people in vulnerable situations, is considered. | |

| | | | |
|---|---|---|---|
| | | g) outlining how it was ensured that vulnerable diversity in potential end-users and other affected people was adequately considered and represented in the participatory evaluation. | |
| 20 | Impacts/Effects of Participation | If applicable, report the methods used to explore the impact of participation as well as barriers and facilitators of participation in the study. | |
| 21 | Usage of AI | Report if AI was used to analyze the data reported in the results section. | |
| 22 | Data Collection & Measurements | Provide details on the data collection methods used for each variable of interest by ...<br>a) describing all relevant aspects of applied data collection methods (e.g., questionnaire, interview, focus groups, observation, task-/scenario-completion, log file analysis).<br>b) explaining which roles established measurements play in the study. If established measurements can't be used, explain why.<br>c) If applicable, adding a detailed description of instrument development and testing (e.g., validity, reliability, feasibility, acceptability, responsiveness, interpretability, appropriateness, precision) of newly designed measurements to the appendix.<br>d) describing the setting of the data collection as well as the dynamics (e.g., laboratory or real setting, participants were grouped into teams, worked collaboratively or individually, predefined tasks to be completed).<br>e) describing the role of the researchers in the evaluation (e.g., supporting role, silent observer). | |
| 23 | Sampling | Describe who participated by ...<br>a) describing any eligibility criteria for participation.<br>b) describing the role and expertise of the participants (e.g., academics, developers, potential end-users, other affected people).<br>c) explaining how it is ensured that the people who were involved were the people who fit the research question (e.g., stakeholder analysis, different digital health literacy levels). | |
| 24 | Recruitment & Engagement Strategies | Describe in detail how participants were recruited, and which strategies were used to engage them by ...<br>a) explaining how participants were selected, e.g., purposive, convenience, consecutive, snowball.<br>b) outlining how participants were approached, e.g., face-to-face, telephone, e-mail.<br>c) describing how, how often, and where the study was announced or advertised. *<br>d) reporting relevant dates, periods of recruitment, and follow-ups. *<br>e) reporting if there were any incentives offered (e.g., monetary or non-monetary incentives such as an offer to provide the study results).<br>f) describing any approaches of engaging participants (e.g., strong and balanced advisory board with clinicians, patients, technical team members able to lead the product design and development, co-creation workshops). Describe formative research and/or content and/or usability testing with target group(s) clearly identified, as appropriate.<br>g) adding to the appendix a description of any material used to support the engagement in the evaluation (e.g., slides to explain concepts, templates to be filled out by participants).<br>h) providing a statement on the science communication strategy.<br>**Note: Fundamentals should be included in the main part. If there is insufficient space, information should be allocated to the appendix.* | |
| | **RESULTS** | | |
| 25 | Main Evaluation Results | Report the most relevant results for the selected and previously described variables of interest and summarize them in the most appropriate total for each methodological step. If applicable, report any conceptual or theoretical developments that have emerged regarding the participation. If applicable, include a relevance or significance analysis of the findings. | |
| 26 | Participants | a) Describe detailed number and expertise of participants involved at each stage of the study (e.g., numbers potentially eligible, examined for eligibility, confirmed eligible, included in the study, completing follow-up, and analyzed).<br>b) Describe the number of drop-outs and their associated context (e.g., how they were responded to, if applicable). | |
| 27 | Participant Checking | Report if participants provided feedback on the findings. | |
| 28 | Selective Reporting | If applicable, to avoid selective reporting and the risk of bias, report if any findings (e.g., negative findings) or unexpected results are not described in detail. Explain why. | |
| 29 | Impacts/Effects of Participation | If applicable, report shortly the measured impact as well as barriers and facilitators of participation at different stages of the study (e.g., development, piloting) | |

| | | | |
|---|---|---|---|
| | **DISCUSSION** | | |
| 30 | Key Findings | Report the key findings of the study and refer to the initial aim of the study. State, what is the (overall) assessment of the intervention. | |
| 31 | Results in Relation to Other Studies | Place your results within the current state of research. State if it supports existing work, expands or challenges other findings, and discuss possible reasons. | |
| 32 | Implications | Explain whether now or in the future, the findings of this study are used to develop or improve a DHI. Highlight the potential influence on digital health design and policy. | |
| 33 | Generalizability | Discuss to what extent the study results could be generalized for other DHIs, populations, or organizations. | |
| 34 | Lessons Learned | Describe any lessons learned from the participatory evaluation of the DHI that could be used to improve future evaluations and outcomes. Reflect on the things that went well and those that did not (e.g., success factors, challenges, or budget considerations). | |
| 35 | Limitations | Acknowledge the limitations of the study, including methodological, ethical, and equity considerations. Address potential impacts on patients and healthcare professionals, policy or practice, and highlight challenges or biases in the participatory approach. | |
| 36 | Future Work | Describe your plans or give recommendations for future research. Refer to the limitations you mentioned, give concrete ideas on how further areas could be examined, or how your results could be integrated in practical implementations. | |
| 37 | Reflecting Participation | Discuss the process and the results of the participation by ...<br>a) commenting if it was possible to carry out the participation as planned. Reflect on the facilitators and barriers for participants to participate in the research and design process.<br>b) describing how participation influenced the study overall. Report (intended or unintended) positive and negative impacts that participation has had on the research, the participants involved (including patients, healthcare professionals, technical team members, and researchers), and wider impacts. Report on any differences the involvement made to the approach or findings of the study.<br>c) reporting the influence of any process or contextual factors that enabled or hindered the impact of participation.<br>d) mentioning barriers or facilitators to the adoption of the intervention among study participants. Relates to individual-level structural, economic, and social barriers or facilitators to access, such as affordability and other factors that may limit a user’s ability to adopt the intervention. | |
| | **OTHERS** | | |
| 38 | Funding | Describe sources of funding and other support. | |
| 39 | Conflict of Interest | In addition to the usual declaration of interests (financial or otherwise), also state the “relation of the study team towards the system being evaluated”, i.e., state if the authors/evaluators are distinct from or identical with the developers/sponsors of the intervention. | |
| 40 | Contributions | Share a detailed description of the contributions of the authors to the published work (e.g., using CRediT). Report if participating citizens or patients or healthcare professionals, or technical team members did co-author and report contributions of team members that are not authors. | |

** Note: Fundamentals should be included in the main part. If there is insufficient space, information should be allocated to the appendix, or a document describing these matters should be cited.*

## References

Moher, David; Schulz, Kenneth F.; Simera, Iveta; Altman, Douglas G. (2010): Guidance for developers of health research reporting guidelines. In: *PLOS Medicine* 7 (2), e1000217. DOI: 10.1371/journal.pmed.1000217.
